# Tuning Interfacial Concentration Enhancement through Dispersion Interactions to Facilitate Heterogeneous Nucleation

**DOI:** 10.1021/acs.jpcc.2c04410

**Published:** 2022-09-16

**Authors:** David McKechnie, Paul A. Mulheran, Jan Sefcik, Karen Johnston

**Affiliations:** † Department of Chemical and Process Engineering, University of Strathclyde, Glasgow G1 1XJ, U.K.; ‡ Doctoral Training Centre in Continuous Manufacturing and Advanced Crystallisation, University of Strathclyde, Glasgow G1 1RD, U.K.; § EPSRC Future Manufacturing Research Hub in Continuous Manufacturing and Advanced Crystallisation, University of Strathclyde, Glasgow G1 1RD, U.K.

## Abstract

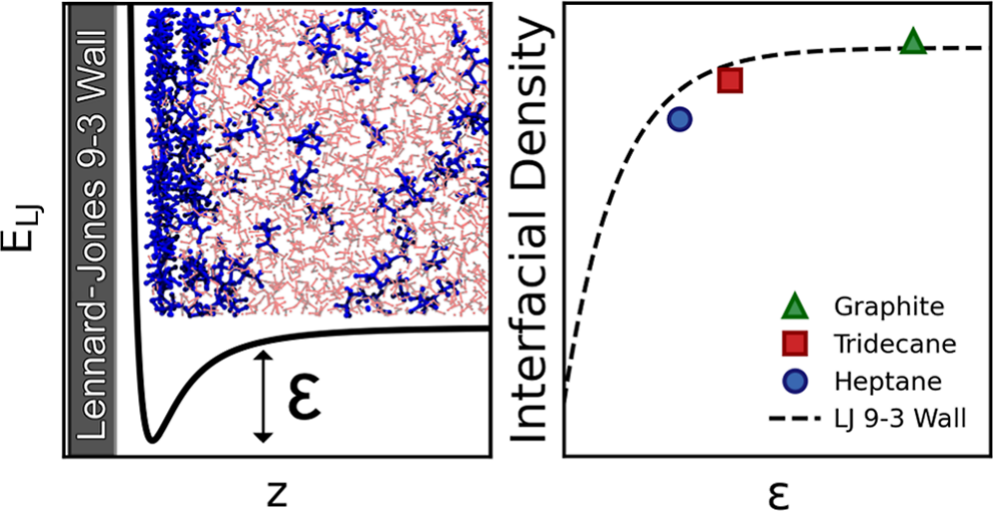

Classical molecular dynamics simulations were used to investigate how dispersion (van der Waals) interactions between non-polar, hydrophobic surfaces and aqueous glycine solutions affect the solution composition, molecular orientation, and dynamics at the interface. Simulations revealed that dispersion interactions lead to a major increase in the concentration of glycine at the interface in comparison with the bulk solution, resulting from a competition between solute and solvent molecules to be or not to be near the interface. This can then lead to kinetic and/or structural effects facilitating heterogeneous nucleation of glycine at non-polar surfaces, in agreement with recent observations for tridecane, graphene, and polytetrafluoroethylene. A novel parameterization process was developed to map a model surface with tunable dispersion interactions to heptane, tridecane, and graphite materials. The model surface was capable of reproducing the solution structure observed in fully atomistic simulations with excellent agreement and also provided good agreement for dynamic properties, at a significantly reduced computational cost. This approach can be used as an effective tool for screening materials for heterogeneous nucleation enhancement or suppression, based on non-specific dispersion interactions based on bulk material molecular properties, rather than interfacial functional groups, templating or confinement effects.

## 1 Introduction

Crystallization from solution can be found in a variety of environmental and biological processes and is also a key purification process in the pharmaceutical, fine chemical, and food industries. It is widely accepted that heterogeneous nucleation is much more prevalent compared to homogeneous nucleation, particularly at low-to-moderate supersaturations and that nucleation from solution largely occurs at interfaces within the system. Several well-known mechanisms that facilitate heterogeneous nucleation include chemical functionalization of the interface,^1^ physical templating,^2^ and confinement.^3−5^

Glycine is the smallest amino acid which is highly soluble in water and has been observed to form mesoscale clusters within aqueous solution.^6,7^ Glycine can be crystallized in three solid forms from aqueous solutions at ambient conditions.^8−11^ In our recent work, we observed an enhanced nucleation rate of glycine at polytetrafluoroethylene (PTFE)^12^ and tridecane^13^ interfaces. These non-polar, hydrophobic interfaces do not fit with the traditional heterogeneous nucleation mechanisms above and therefore would not be expected to enhance heterogeneous nucleation of highly polar, hydrophilic glycine.

However, hydrophobic interfaces, such as oils or polymers, are frequently present in nucleation experiments, including microfluidics,^14,15^ millifluidics,^16^ microwells,^17^ polymer tubings, and stirrer coatings.^12^ Therefore, it is important to understand how such interfaces can facilitate heterogeneous nucleation of highly polar, hydrophilic molecules from solution. Classical molecular dynamics (MD) simulations reveal an enhanced glycine concentration at the tridecane–solution interface and it was proposed that the concentration enhancement is due to non-specific van der Waals interactions between the interface and the solution.^13^ The local concentration enhancement at the interfaces can then lead to kinetic and/or structural effects facilitating glycine nucleation.

In this work, we have performed MD simulations of aqueous glycine solutions with a model surface with tunable dispersion interactions represented by a Lennard-Jones (LJ) 9-3 wall potential. This has allowed us to investigate the effect of surface–solution dispersion interactions on the solution composition, molecular orientation, and dynamics within the interfacial region in comparison with the bulk solution. The model surface wall potential reflects van der Waals interactions between solution molecules and the surface and has been parameterized to represent specific materials (heptane, tridecane, and graphite). We show that the concentration enhancement effect observed in simulations with full molecular description of these materials can be reproduced using a simple wall potential, at a significantly reduced computational cost. We also examine the effect of electrostatics and their significance compared to those observed for dispersion interactions. This work presents a novel methodology that provides insights into how specific materials are expected to influence the interfacial solution region and facilitate heterogeneous nucleation and can therefore be used in future work as a design tool for materials’ selection for the purposes of nucleation control.

## 2 Methodology

In this section, we provide the details of the force fields used to model the glycine and water molecules within the solution. We then give a brief description of the model surface and the combining rules used to determine the interactions between the surface and the solution atoms within the simulations. Following this, we explain the parameterization procedure that was developed to determine suitable LJ parameters to allow real materials to be represented by the LJ wall. Finally, we describe the system setup and give MD simulation details for the glycine solution in contact with the model and atomistic surfaces to determine the effects of the dispersion and electrostatic interactions.

### 2.1 Force Fields

For glycine, we used the generalized AMBER Force Field (GAFF)^18^ with CNDO charges,^19^ which was found to give the best results for crystalline α-glycine, glycine solutions, and α-glycine in contact with a supersaturated solution.^20^ The SPC/E water model^21^ was used as recommended in the previous study as it was found to accurately represent the density and diffusion coefficients within the system. This combination of glycine and water models has also been shown to accurately reproduce the solubility of α-glycine for temperatures close to 298 K.^22^ For tridecane, we used the AMBER-ii force field that was developed for alkanes by Nikitin et al.^23^ Graphite and PTFE were also modeled using GAFF. 1-4 interactions (interactions between atoms separated by three consecutive bonds) and were reduced to 0.5 and 0.8333 for LJ and electrostatics, respectively, as intended for AMBER style force fields. The force field parameters are given in the Supporting Information.

### 2.2 System Setup and MD Simulations

MD simulations were performed using LAMMPS^24^ in the NVT ensemble. The temperature was maintained at 298 K using a Nosé–Hoover thermostat with a damping parameter of 0.1 ps. All simulations were performed using a time step of 2.0 fs.

**Figure 1:**
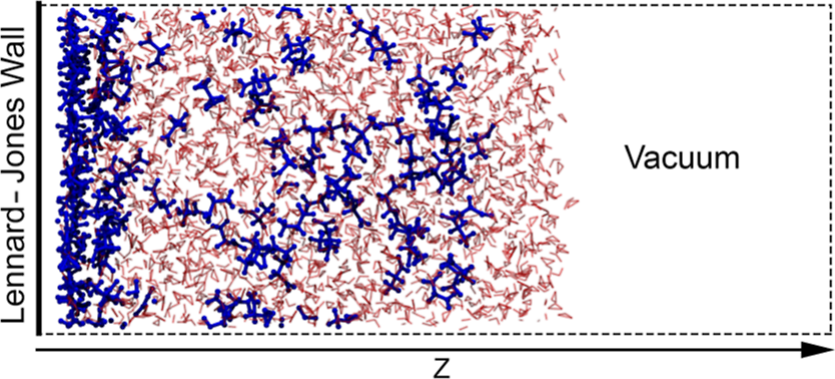
Schematic of the simulation setup. Glycine and water molecules are shown in blue and red, respectively. The dashed black lines represent the boundaries of the simulation box, while the thick black line shows the position of the LJ wall. System snapshot was generated using VMD.^26^

Periodic boundary conditions were applied in the *x* and *y* directions, and non-periodic boundaries in the *z* direction. Each simulation box was large enough in the *z* direction to maintain a vacuum layer on one side of the glycine solution to prevent compression of the glycine solution against the solid surface, as shown in the schematic in Figure 1. LJ interactions, including the LJ 9-3 wall potentials, were truncated at a cutoff of 1.4 nm. Short-range electrostatics were calculated below 0.98 nm and long-range electrostatics were calculated using a particle–particle particle-mesh with an accuracy of 1 × 10^–6^. A slab correction was included to allow for long-range electrostatics to be applied that treats the system as if it was periodic in *z*, but with an empty volume inserted between the slabs, effectively removing the electrostatic interactions between the slabs.^25^

Glycine solution films were prepared with a range of thicknesses for concentrations of 296.7 and 500.7 g/kg (where g/kg refers to g_glycine_/kg_water_), which are both supersaturated with respect to the solubilities of both α-glycine and γ-glycine at 298 K which are 247.2 and 228.6 g/kg, respectively.^11^ The *x*–*y* cross-sectional area for each film was 3.45 × 3.45 nm^2^ and film thicknesses ranged from 3.1 to 13.9 nm. Details of the number of molecules in each film and the equilibration and production simulation times are given in the Supporting Information.

In order to determine the effects of the surfaces on the dynamics of the glycine molecules within the solution, a 296.7 g/kg solution was simulated with periodic boundaries in all directions using the NPT ensemble. The temperature was maintained at 298 K using the same thermostat parameters described above, and the pressure was maintained at 1 atm using a Nosé–Hoover–Andersen barostat with a damping parameter of 1 ps.

To determine the impact of electrostatics on the solution composition at the interface, we performed some further simulations of the 3.1 nm thick, 296.7 g/kg solution film in contact with an atomistic, crystalline tridecane surface with varying charge sets and LJ parameters. LJ parameters for PTFE from GAFF^18^ and tridecane from AMBER-ii^23^ were used to vary the dispersion interactions. Three charge sets were used to vary the electrostatic interactions: charges for tridecane from AMBER-ii, charges for PTFE from GAFF, and the PTFE charges from GAFF doubled. The LJ parameters and charges are included in the Supporting Information.

Thermodynamic properties were sampled every 200 fs, whereas structural and dynamic properties were sampled every 10 ps. The structural property profiles, such as density and molecular orientation, were analyzed as a function of the distance from the interfaces. Interfacial regions were defined by taking a 1 nm zone from the point at which the LJ 9-3 potential crosses the *x*-axis (*z* = 0.715σ_ww_) to prevent differences in the exclusion zone of various LJ 9-3 walls influencing the results. The translational mobility and residence time of the glycine molecules within the layers formed in the interfacial region were investigated by calculating the mean squared displacement (MSD), using multi-time origin analysis, in the *x* and *y* directions (parallel to the surface), while the molecules remain in the interfacial region. The rotational mobility was analyzed by calculating the autocorrelation function (ACF) with multi-time origin of the C–C bond vector of the glycine molecules while they remain in the interfacial region. The mobility within the interfacial region has been compared to the mobility of glycine in a fully periodic, bulk simulation of glycine solution at the same concentration. Residence times have been compared with slices of equal size in each case to provide an equal comparison.

### 2.3 Model Interface and Mixing Rules

An LJ 9-3 potential can be obtained by integrating over a 3D half lattice of LJ particles interacting via a 12-6 potential.^27^ Other functional forms of wall potentials do exist, such as the LJ 10-4 potential, which can be obtained by integrating over the surface plane of the 3D lattice of LJ 12-6 particles. As the interactions are between the solute and an oil layer, the LJ 9-3 potential is most appropriate.

The functional form of the LJ 9-3 potential implemented within the LAMMPS code is given by
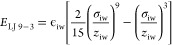
1where ϵ_iw_ controls the depth of the well of the interaction between the wall w and a given atom i, σ_iw_ is related to the distance at which the potential crosses the *x*-axis, and *z* is the perpendicular distance from the wall to the atom. The wall potential allows the interface to be represented without requiring the interactions for each atom within the interface to be calculated explicitly, resulting in a significantly reduced computational cost. It also provides a framework in which to predict the interfacial concentration enhancement of the solute induced by a new material, without requiring expensive and time-consuming atomistic MD simulations.

**Figure 2:**
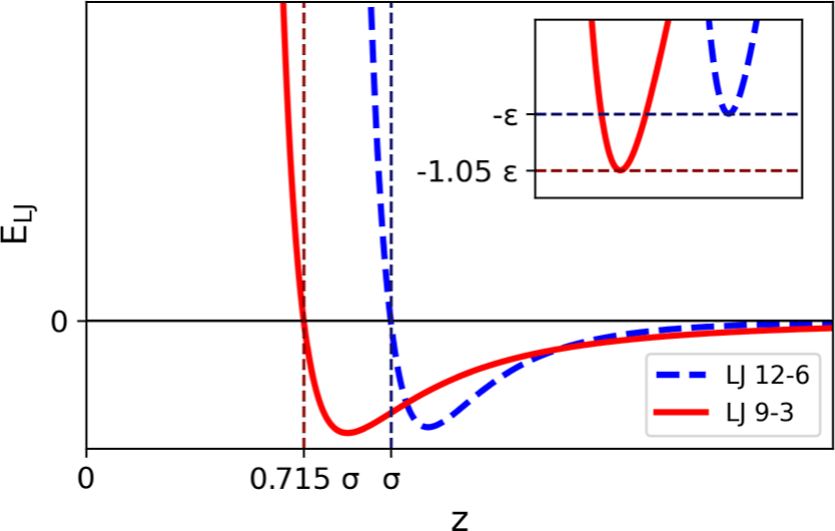
LJ 9-3 and 12-6 potentials of the same ϵ and σ parameters. The dashed vertical lines show the point at which the potential crosses the *x*-axis. The inset shows the region close to the potential well. The horizontal dashed lines show the position of the minimum of the well.

It is important to note that in eq 1 although ϵ and σ play a similar role to the standard LJ 12-6 potential, they correspond to different points on the potential which has implications on the use of combining rules to produce the ϵ_iw_ and σ_iw_ parameters. To demonstrate this, Figure 2 shows LJ 12-6 and 9-3 potentials of the same ϵ and σ parameters. The LJ 12-6 interaction crosses the *x*-axis at a distance equal to σ, whereas the LJ 9-3 interaction crosses the *x*-axis at 0.715σ.

Lorentz–Berthelot rules are frequently used to produce ϵ and σ parameters for interactions between different particle types. The arithmetic mean is used to combine σ parameters, however, due to the different position of σ in eq 1 we used the adjusted eq 2
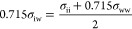
2

The geometric mean is used to combine ϵ parameters and as there is only a 5% difference between the LJ 9-3 and 12-6 potential well depths, as seen in Figure 2, we have used the standard geometric mean combining rule
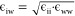
3

### 2.4 Model Interface Parameterization

Although it is possible in MD simulations to model a variety of interfacial materials atomistically, these simulations can be computationally expensive. Wall potentials, where the interaction depends only on the distance from the wall, can significantly reduce the number of interactions between particles and thus reduce the computational cost of the simulations. Furthermore, once a suite of wall potential simulations have been performed, there is no need for further atomistic MD simulations, provided the material of interest can be reliably mapped onto its corresponding wall potential, and the solution remains unchanged.

In order to relate the wall potential to specific materials, it is necessary to map the wall potential parameters to those of the atomistic representation. We have developed a procedure to parameterize ϵ_ww_ and σ_ww_ values to produce wall potentials that represent specific materials, and this has been applied to heptane, tridecane, and graphite. In each case, a slab of the material was created so that it was at least 3 nm in the *x*, *y*, and *z* directions. To remove effects related to changes in the interface height (such as due to capillary waves) and surface variation, flat surfaces with periodic structures were prepared. For heptane and tridecane, pseudo-crystalline structures of periodic extended chains lying parallel to the interface were prepared, so that the densities matched the experimental densities of the liquids at 298 K. For graphite, the crystal structure was used directly. A snapshot of the crystalline tridecane slab is shown, with its dimensions, as an example in the Supporting Information.

In order to derive suitable wall parameters to represent a given surface, we first estimated ϵ_iw_ and σ_iw_ between the atoms within the solution and the surface. These parameters can then be used to derive the ϵ_ww_ and σ_ww_ parameters that best represent the surface by using the combining rules eqs 2 and 3. This was achieved by breaking the molecules down to their constituent atoms and mapping their interactions with the surface. The interactions were mapped for each of the atom types within glycine and the oxygen atom within water. The hydrogen atoms of the water molecules could not be included within the parameterization process as they do not have LJ interactions with any other atoms. To map the interactions, an individual atom was placed at a set distance, *z*, from the surface and the total LJ 12-6 interaction between the atom and the surface was calculated. The distance *z* was then increased by a small amount and the process was repeated in order to map out the *z*-dependent LJ potential between the surface and the atom. Equation 1 was then fit to these values to obtain suitable ϵ_iw_ and σ_iw_ parameters.

The LJ 9-3 potential is uniform along the interface plane and does not represent the variation in potential at different lattice sites.^28^ To account for this, the parameterization process was repeated at 36 individual *x*, *y* positions across the surface and the average ϵ_iw_ and σ_iw_ was taken. A detailed example of the parameterization of the LJ interaction between tridecane and the nitrogen atom of glycine is provided in the Supporting Information for clarity.

This process was repeated for each atom type in order to build a full set of ϵ_iw_ and σ_iw_ parameters that represent the interaction between the surface and the solution. The obtained ϵ_iw_ and σ_iw_ parameters can be plotted against the LJ parameters of the individual atoms, ϵ_ii_ and σ_ii_. Equations 2 and 3 are then fit to this data in order to obtain suitable parameters that describe the surface, ϵ_ww_ and σ_ww_, as presented in the Results and Discussion section. These wall parameters can then be combined with any given atom, with parameters ϵ_ii_ and σ_ii_, using the above combining rules to obtain suitable parameters that describe the surface–atom interaction.

## 3 Results and Discussion

In this section, we show how dispersion and electrostatic interactions influence the concentration, orientation, and dynamics of glycine in the interfacial region and discuss implications of the differences between the interfacial region and the bulk solution for the heterogeneous nucleation of glycine. In Section 3.1, we first discuss the parameterization of the model interface for the interaction of glycine solution with heptane, tridecane, and graphite and then validate our parameterization approach by comparing the solution behavior at the LJ wall for tridecane and at atomistic tridecane. In Section 3.2, we explore a range of wall parameters spanning the values of the three materials. In Section 3.3, we investigate how finite size effects influence the results of the MD simulations. In Section 3.4, we examine the effect of electrostatic interactions and their relative impact in comparison to dispersion interactions. Finally, in Section 3.5 we discuss how these insights can help identification of materials that would facilitate heterogeneous nucleation of glycine.

### 3.1 Model Interfaces for Tridecane, Heptane, and Graphite

**Figure 3:**
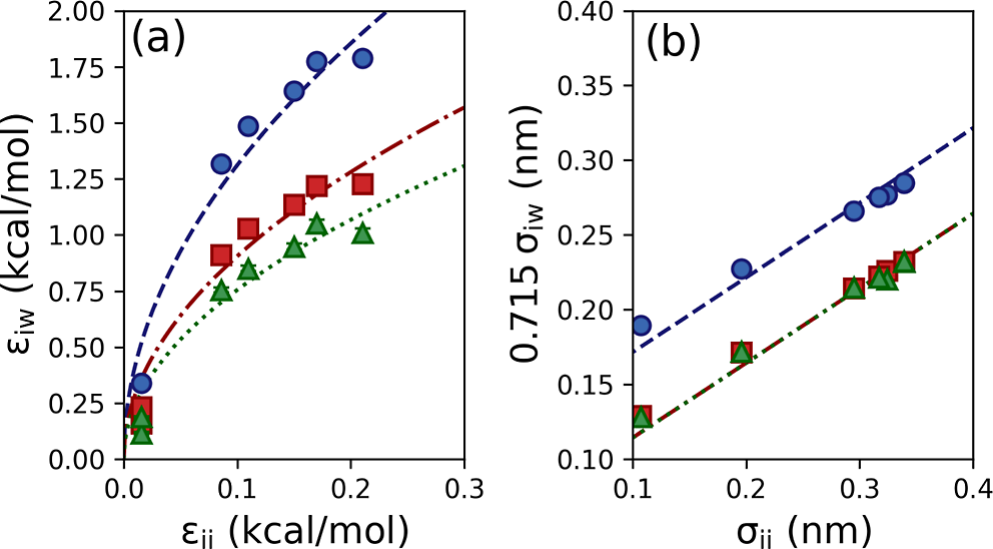
(a) ϵ_iw_ and (b) 0.715σ_iw_ parameters for the interactions of each atom type and graphite (blue circles), tridecane (red squares), and heptane (green triangles) plotted against their equivalent atom–atom parameters. Error bars represent the standard error, but in most cases are smaller than the markers. Dashed and dotted lines represent fits to eqs 2 and 3.

The parameterization process described in Section 2.4 was performed for each of the glycine atom types and the water oxygen atom in combination with the tridecane, heptane, and graphite surfaces. This allowed ϵ_iw_ versus ϵ_ii_ and σ_iw_ versus σ_ii_ plots to be constructed for each of the three interfacial materials as shown in Figure 3, where each point represents the average ϵ_iw_, or σ_iw_, obtained from the 36 *x*–*y* positions across the surface. Equations 2 and 3 were then fit to each of these data sets to obtain the ϵ_ww_ and σ_ww_ parameters that best represent each of the interfacial materials. The dashed lines in Figure 3 represent these fits and the resulting wall parameters are provided in Table 1. Good fits are obtained for each set of data. It can be seen that at the lowest ϵ_ii_ and σ_ii_ parameters the fits overestimate the obtained values. As these values correspond to the hydrogen atoms of the glycine molecules, this deviation is not expected to significantly impact the behavior of the system. In terms of wall strength, heptane has the weakest interaction, followed by tridecane and finally graphite. As the graphite interface provides an ideal flat surface, the parameterization process returns a σ_ww_ value that is equal to σ_cc_ of the carbon atoms within the interface. We note that for the heptane and tridecane interfaces, the σ_ww_ values obtained are lower than the σ_cc_ and σ_hh_ parameters of the atoms within the interface due to the more corrugated nature of the surface.

**Table 1:** Fitted ϵ_ww_ and σ_ww_ Parameters for Selected Materials

material	ϵ_ww_ (kcal/mol)	σ_ww_ (nm)
heptane	5.7	0.18
tridecane	8.2	0.18
graphite	17.2	0.34

**Figure 4:**
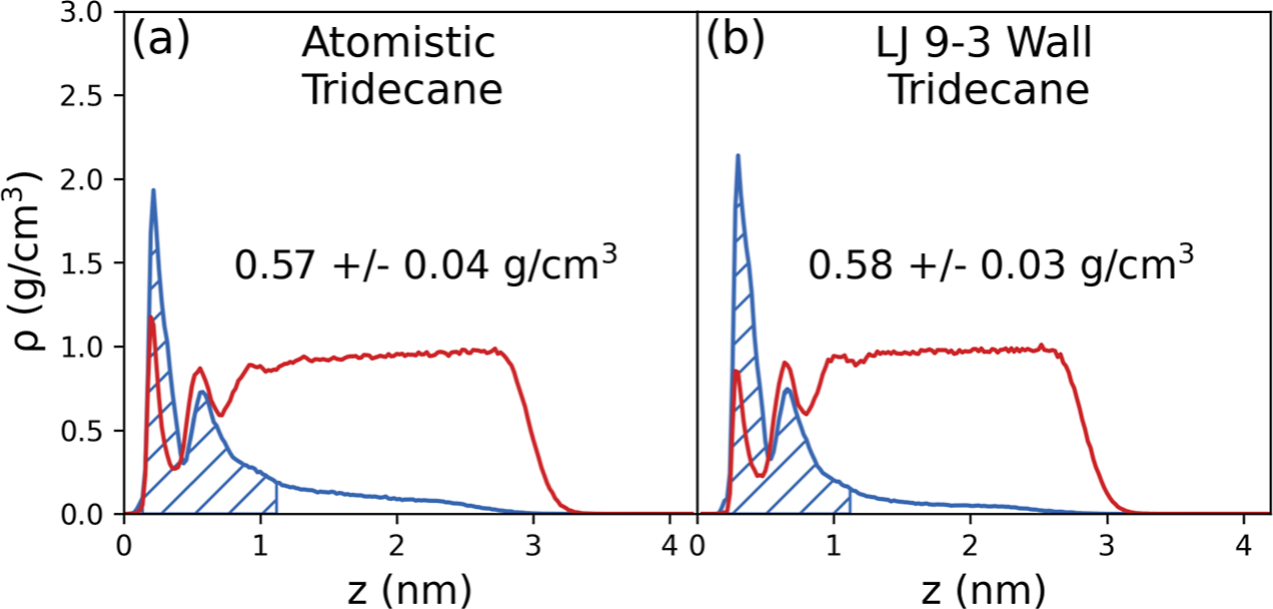
Density profiles for glycine (blue) and water (red) obtained for glycine solution in contact with (a) atomistic crystalline tridecane and (b) the corresponding LJ 9-3 wall. The hatched area indicates the 1 nm interfacial region.

To validate the parameterization of the model surfaces, the properties of the 3.1 nm thick, 296.7 g/kg film in contact with the atomistic, crystalline tridecane surface and the LJ 9-3 wall parameterized for tridecane were compared. The density profiles obtained for each simulation are shown in Figure 4. There is excellent agreement between the density profiles, with density oscillations of similar magnitude in both cases, although the LJ 9-3 wall predicts slightly lower water density in the peak closest to the interface.

We are particularly interested in the concentration of glycine within the interfacial region. We have defined the interfacial region as a 1 nm zone from the point at which the LJ 9-3 potential crosses the *x*-axis (*z* = 0.715σ_ww_) to ensure that the increasing size of the exclusion zone for increasing values of σ_ww_ did not influence the obtained results. The 1 nm region is sufficiently wide to capture the layered section of the film. The average glycine densities within the interfacial region, denoted as ρ_int_, of the atomistic tridecane and LJ 9-3 wall are 0.57 and 0.58 g/cm^3^, respectively, showing that the LJ 9-3 wall is able to accurately reproduce the glycine interfacial density.

**Figure 5:**
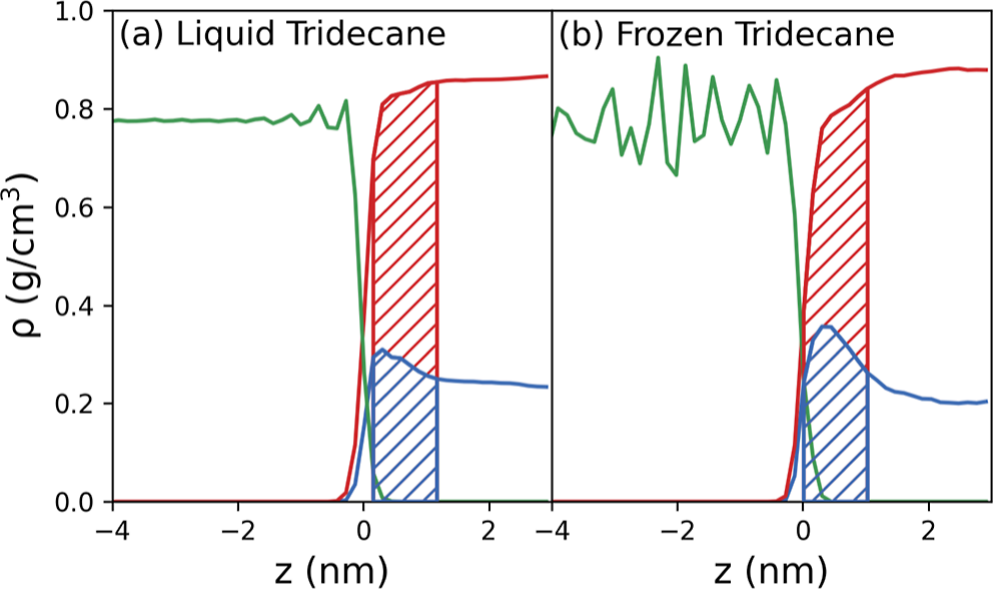
Density profiles obtained for glycine solution films with a concentration of 307 g/kg in contact with (a) fully liquid tridecane and (b) frozen, amorphous tridecane. The hatched area represents the defined 1 nm interfacial region. The point at which the tridecane and water density profiles cross is set to *z* = 0.

The layering seen in the density profiles in Figure 4 was not observed in the previous simulations of the glycine solution at a mobile, liquid–liquid tridecane–solution interface.^13^ This layering is due to the fixed, flat nature of the interfaces in the present work. Our analysis of density profiles does not account for capillary waves and interfacial fluctuations that are present in the liquid–liquid interface and as such the density at the fluctuating surface is smeared. It is possible that the layering would become clearer using more advanced interfacial analysis techniques, such as the generalized identification algorithm for non-planar interfaces.^29^ To provide further insights into the effects of liquid–liquid versus solid–liquid interfaces on the obtained density profiles, a simulation of a film of glycine solution at 307 g/kg (the same solution that was simulated in the previous work^13^) was carried out in contact with frozen liquid tridecane (an amorphous, solid surface). This frozen, amorphous surface acts as an intermediate step between the fully liquid and crystalline tridecane surfaces. The density profiles for the liquid and frozen surfaces are shown in Figure 5. The layering is not present in either case due to the uneven surface, although the glycine density peak is slightly higher and sharper for the frozen tridecane compared to the liquid tridecane.

**Figure 6:**
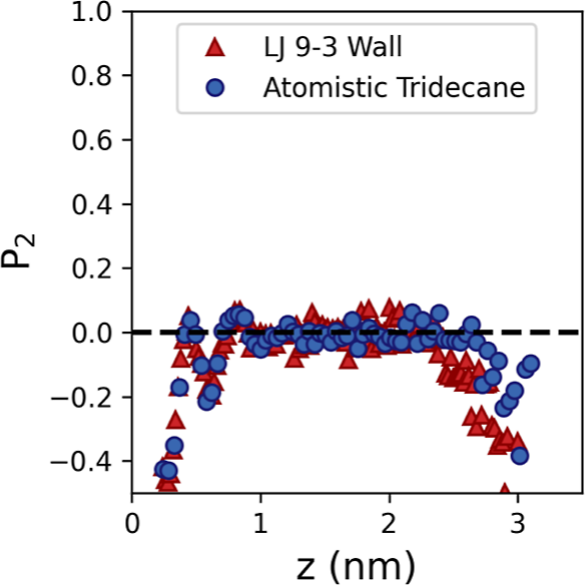
Bond order profiles for glycine solution in contact with the atomistic crystalline tridecane (blue circles) and the LJ 9-3 wall fitted to represent the crystalline tridecane (red triangles).

We further compare the structures of the solution at the LJ 9-3 wall and at the atomistic tridecane surface by analyzing the orientation of the glycine molecules using the bond orientation parameter *P*_2_, which is defined as
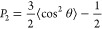
4where θ is the angle between the *z*-axis and the C–C bond vector. A *P*_2_ value of 1.0 corresponds to the C–C bond being orientated perpendicular to the surface, whereas a *P*_2_ value of −0.5 indicates that the bond is lying parallel to the surface. If the molecules are randomly oriented, as would be expected in bulk solution, the *P*_2_ value will average to 0. The *P*_2_ values for the atomistic tridecane and LJ 9-3 tridecane simulations are shown in Figure 6. Both simulations demonstrate the expected random orientation of glycine in the bulk of the film, and ordering at both the solid–solution and solution–vacuum interfaces. At the solid–solution interface, we see a strong alignment of glycine with the C–C bond parallel to the surface in the first layer and a weaker alignment within the second layer. The position and magnitude of the peaks in the *P*_2_ values show excellent agreement between the two simulated systems, demonstrating that the LJ 9-3 wall successfully matches the structural details of the fully atomistic interface. We see stronger alignment with the solid surfaces than was observed in the previous liquid tridecane simulations,^13^ suggesting that the mobile liquid–liquid tridecane–solution interfaces also obscure, or disrupt, the ordering of the molecules.

**Figure 7:**
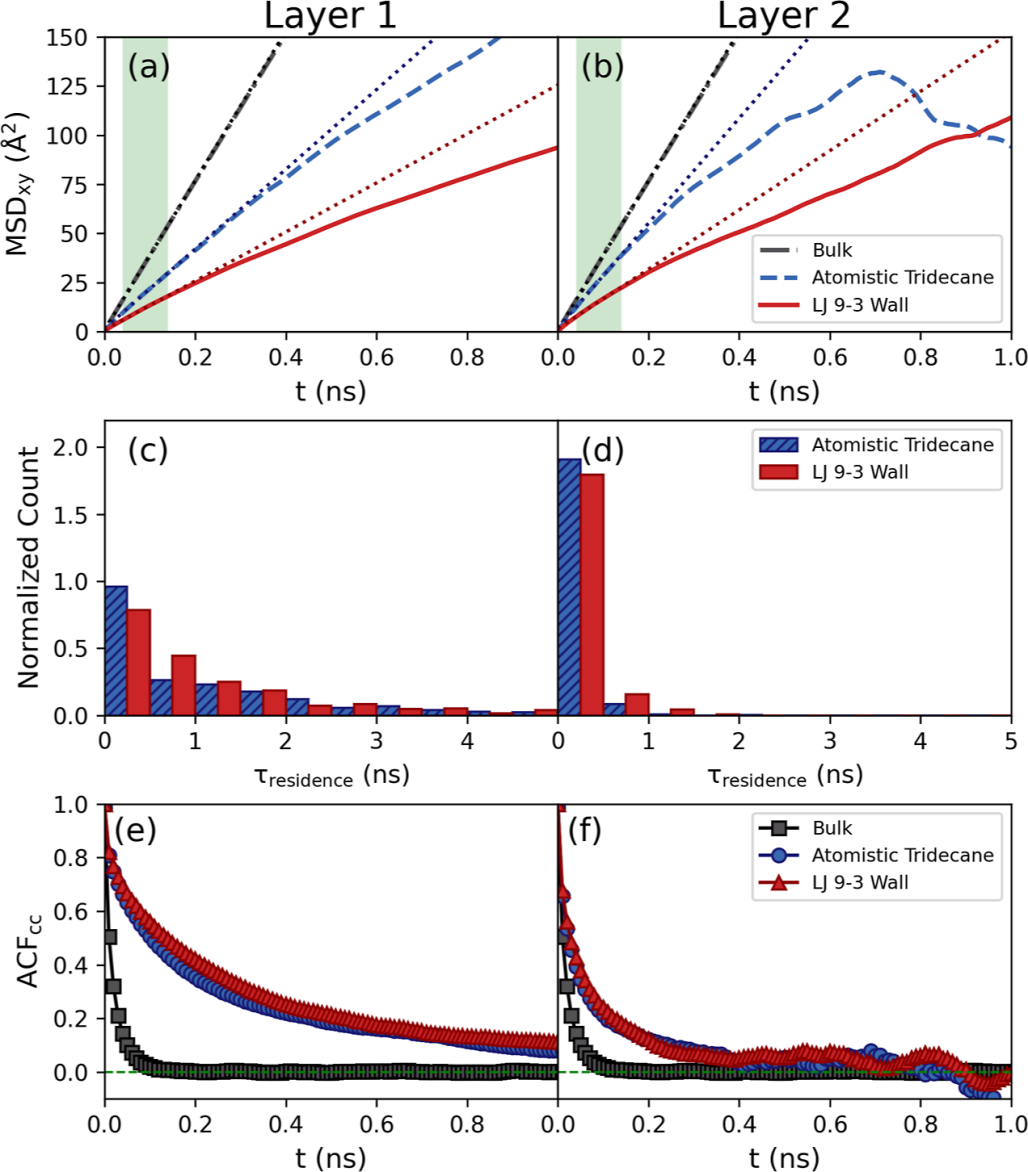
Comparison of dynamic properties of glycine molecules in contact with the atomistic tridecane surface, the LJ 9-3 wall representing tridecane, and bulk solution at the same overall concentration (296.7 g/kg). The top row shows MSD_*xy*_ and the green area indicates the region used for the linear fit (dotted lines) used to obtain the diffusion coefficients. The second row shows the residence times of glycine molecules in the first and second interfacial layers. The third row shows the ACF of the C–C bond orientation in the first and second layers compared to bulk solution. Graphs on the left correspond to the first layer, whereas graphs on the right correspond to the second layer.

The translational and rotational mobility of the glycine molecules within the two layers that form at the interface was also investigated. The first interfacial layer was defined for the LJ 9-3 surface as the region between 0.715σ_ww_ and the point of minimum glycine density between the first and second peaks in the glycine density profile. This gave the first interfacial layer a thickness of 0.41 nm and an equally sized region directly following this was taken as the second interfacial layer. The same layers were used for analysis in the atomistic tridecane interface simulation to allow for a fair comparison. The translational mobility parallel to the surface was determined using the two-dimensional MSD of the glycine molecules in the *xy* plane while they remain in a given layer and is denoted as MSD_*xy*_. Figure 7a,b shows MSD_*xy*_ for the glycine molecules at both the atomistic tridecane and LJ 9-3 tridecane surfaces together with MSD_*xy*_ for the bulk glycine solution at the same concentration.

For both interface types, MSD_*xy*_ was slower in the layers compared to the bulk solution. MSD_*xy*_ in the two interfacial layers is similar, with the layer closest to the surface being marginally slower. MSD_*xy*_ was higher for the atomistic interface than for the LJ 9-3 wall. It may be expected that the LJ 9-3 wall would result in faster dynamics along the surface due to reduced friction as has been seen previously for simulations of water in contact with a LJ 9-3 wall.^30,31^ This behavior could be a result of the higher glycine concentration at the LJ 9-3 wall, as the diffusion coefficient of glycine in aqueous solution is known to decrease with increasing glycine concentration.^32^

The diffusion coefficient of the glycine molecules parallel to the surface were used to further compare the dynamics between the different interfaces. The diffusion coefficient, *D*, were obtained from MSD_*xy*_ using the Einstein relation
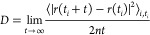
5where *t* is the time interval, *r*(*t*_*i*_) is the position of the molecule at time *i*, and *n* is the number of dimensions that are being analyzed (in this case *n* = 2). The diffusion is averaged over all molecules *i* within the interfacial region at each time step output to the trajectory.

A region close to *t* = 0 where the MSDs are quite linear, highlighted by the green shaded area as shown in Figure 10a,b, was used to determine *D*. For the bulk solution, we obtain *D* = (0.4689 ± 0.0004) × 10^–9^ m^2^ s^–1^. For comparison, the experimentally obtained *D* is ≈0.63 × 10^–9^ m^2^ s^–1^ for similar concentrations.^32^ The values of *D* in each layer for both the LJ 9-3 and atomistic surfaces are given in Table 2. *D* for the LJ 9-3 wall are approximately 40% lower than those obtained for the fully atomistic tridecane surface.

**Table 2:** Diffusion Coefficients, *D*_*xy*_, for Movement Parallel to the Surface for Glycine Molecules within the First and Second Interfacial Layers^a^

	*D* (× 10^–9^ m^2^ s^–1^)	
interface type	layer 1	layer 2
atomistic tridecane	0.253 ± 0.001	0.332 ± 0.003
LJ 9-3 tridecane	0.154 ± 0.001	0.187 ± 0.002

a Errors are the standard error of the estimated slope.

The residence time of the molecules within each layer was analyzed to provide information about the molecules’ mobility perpendicular to the surface. The distribution of residence times for the glycine molecules in the interfacial layers for the atomistic tridecane and LJ 9-3 tridecane surfaces are shown in Figure 7c,d. There is good agreement between residence times obtained for the atomistic tridecane and the LJ 9-3 tridecane surfaces, demonstrating that the LJ 9-3 wall is accurately reproducing the mobility of the glycine molecules perpendicular to the surface. The residence times for the first interfacial layer are longer than for the second layer, which is consistent with the slower dynamics observed in the first layer.

Finally, the rotational mobility of the molecules was analyzed using the autocorrelation function of the C–C bond vector, denoted as ACF_cc_, of the glycine molecules while they remain in an interfacial layer. ACF_cc_ is given by
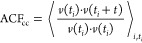
6where *t* is the time interval and *v*(*t*_*i*_) is the bond vector at times *i*. The ACF_cc_ is averaged over each molecule *i* within the interfacial region at each time step output to the trajectory. Figure 7e,f shows ACF_cc_ for the atomistic tridecane and LJ 9-3 wall and for the bulk solution at the same concentration. There is excellent agreement between the atomistic tridecane and LJ 9-3 wall, and once again we see slower dynamics in the interfacial region compared to the bulk solution.

To quantify the effects of the interfaces on the rotational mobility of the glycine molecules, we defined a decay time as the time it takes for the autocorrelation function to decrease to 1/*e*. The decay times for the interfacial layers for the atomistic tridecane and LJ 9-3 wall are given in Table 3, and they are in reasonable agreement. The decay time for the bulk glycine solution is 0.015 ± 0.005 ns, which is much faster than the decay times in interfacial layers, as expected.

**Table 3:** Decay Times for the ACF of the C–C Bond of Glycine Molecules within the First and Second Interfacial Layers

	decay time (ns)	
interface type	layer 1	layer 2
atomistic tridecane	0.185 ± 0.005	0.045 ± 0.005
LJ 9-3 tridecane	0.255 ± 0.005	0.055 ± 0.005

Overall, we see good agreement between the dynamics of the glycine molecules at the atomistic tridecane and LJ 9-3 wall interfaces, with both systems demonstrating slower dynamics within the interfacial layers compared to the bulk glycine solution at the same concentration. These results demonstrate that the LJ 9-3 wall accurately reproduces the solution structure and dynamics at the tridecane interface. Furthermore, the LJ 9-3 wall simulations have a significantly lower computational cost compared to the atomistic interface. Using 10 cores of an Intel Xeon Gold 6138 (Skylake) processor, the atomistic tridecane surface simulation completed 5.71 ns/day, whereas the LJ 9-3 wall simulation completed 10.19 ns/day, a performance increase of 78%. Although the performance increase will depend on a number of factors, such as the hardware used or the ratio of atoms between the interface and the solution, this clearly demonstrates that the LJ 9-3 wall provides a significant reduction in the computational cost.

### 3.2 Tuning the Interfacial Concentration

**Figure 8:**
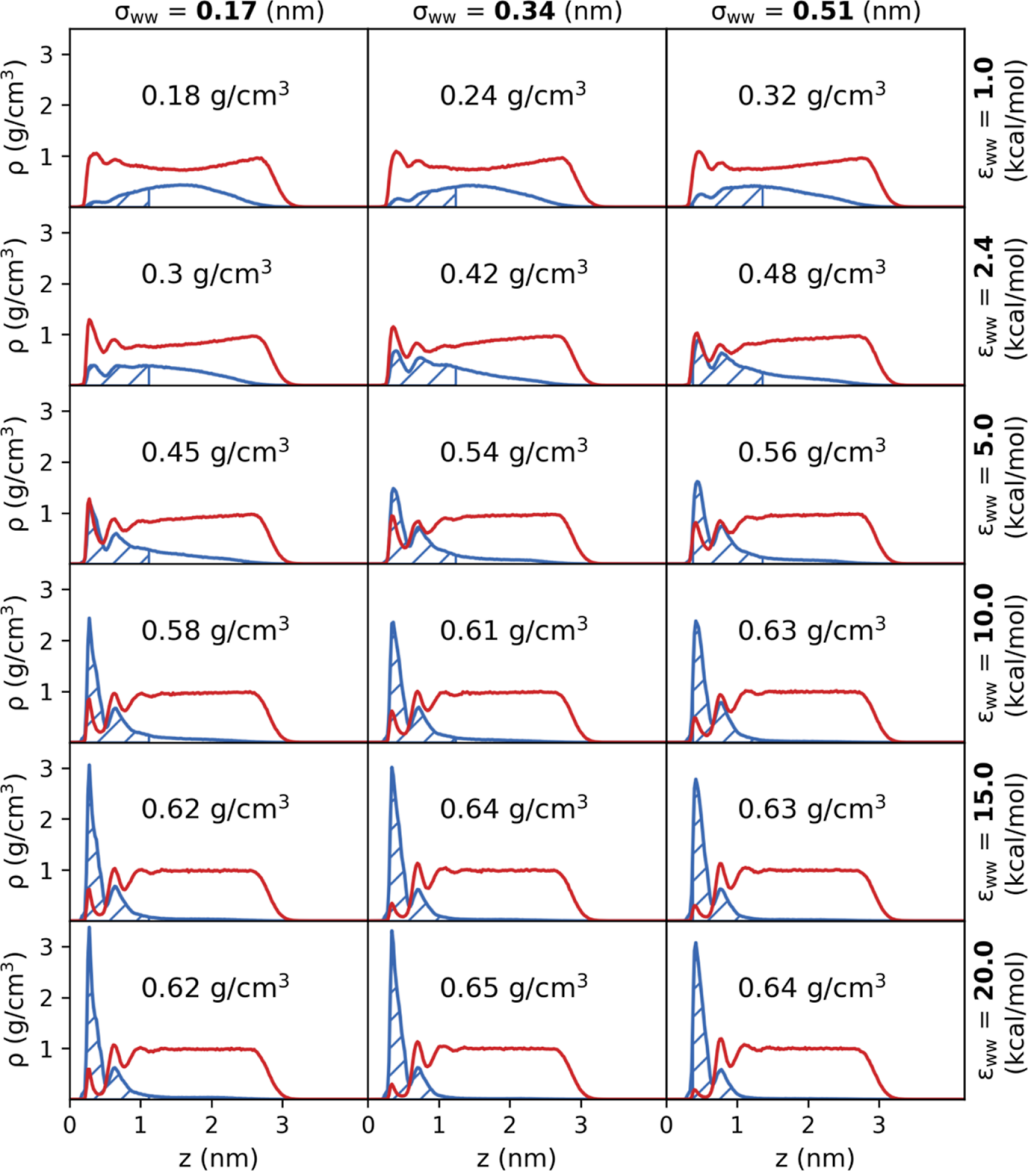
Density profiles for glycine (blue) and water (red) for solutions in contact with a LJ 9-3 wall with varying ϵ and σ parameters. The hatched area under the glycine profile indicates the 1 nm region defined as interfacial. The glycine density within the interfacial region is given for each profile.

We will now explore a range of wall parameters to show how they can be used to tune the interfacial concentration of glycine. We have carried out simulations of the 3.1 nm thick, 296.7 g/kg film in contact with LJ 9-3 walls for each combination of ϵ_ww_ values 1, 2.4, 5, 10, 15, and 20 kcal/mol and σ_ww_ values of 0.17, 0.34, and 0.51 nm. This parameter range spans the parameters of the three materials, namely heptane, tridecane, and graphite, which were presented in Table 1. The resulting density profiles in aqueous glycine solutions for the LJ wall parameters are shown in Figure 8.

As previously seen in Section 3.1, there is significant layering of the solution at the LJ 9-3 wall. As ϵ_ww_ increases, the layering of glycine at the interface increases significantly. Increasing σ_ww_ also results in an increase in the interfacial concentration of glycine, however, this effect is much less pronounced than for ϵ_ww_. For values of ϵ_ww_ greater than 2.4 kcal/mol, a highly dense layer of glycine forms at the interface, and at the highest values of ϵ_ww_ the amount of water at the interface is reduced as the glycine becomes more concentrated.

**Figure 9:**
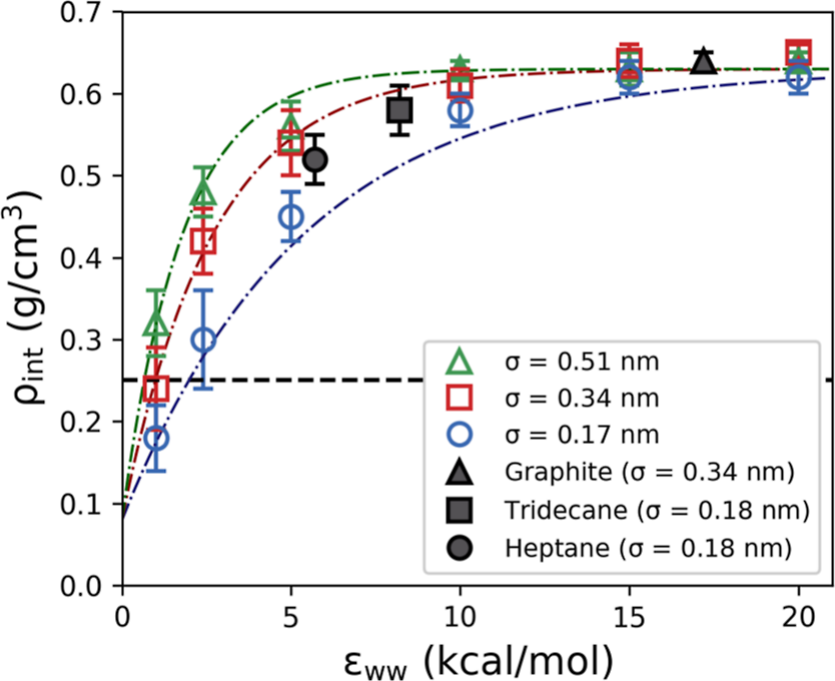
Interfacial glycine densities of the LJ 9-3 walls fitted to the realistic materials (black markers) plotted with the densities of the grid of LJ 9-3 parameter simulations (open, colored markers). Colored dot-dashed lines are fits to eq 7 for σ_ww_ values of 0.17, 0.34, and 0.51 nm. The dashed black line shows the density of glycine for a bulk-like solution of the same overall concentration.

The variation of ρ_int_ for each combination of wall parameters is shown in Figure 9. It is clear that ϵ_ww_ has a strong influence on the interfacial density, with the values plateauing beyond an ϵ_ww_ value of 10 kcal/mol as the bulk of the film becomes increasingly depleted. The interfacial densities obtained using the walls parameterized for heptane, tridecane, and graphite are also included in Figure 9 and can be seen to follow the trends set by the other points. This means that the interfacial densities induced by other materials can be estimated from the corresponding wall parameters obtained, allowing materials to be screened at a greatly reduced computational cost.

The values from the simulation grid were fit to an asymptotic regression model
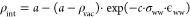
7where ρ_int_ is the interfacial density, *a* is a fitting parameter that corresponds to the maximum interfacial density for the current film size, ρ_vac_ is the interfacial density for the glycine solution film in contact with vacuum, and *c* is a fitting parameter that is proportional to the rate at which the interfacial density increases with ϵ. The resulting functions for σ_ww_ = 0.17, 0.34, and 0.51 nm are shown as the dotted lines in Figure 9. Although it is possible to simulate the glycine solution film in contact with vacuum on each side, it is unclear how the 1 nm interfacial region could be defined that is consistent with the LJ 9-3 wall simulations due to the nature of the vacuum–solution interface. As such we have chosen to include ρ_vac_ as a fitting parameter. From the fit to the simulation grid data, we obtain *a*, ρ_vac_, and *c* parameters of 0.63 g/cm^3^, 0.08 g/cm^3^, and 0.11 mol kcal^–1^ nm^–1^, respectively. We can see that we obtain a good fit for σ_ww_ values of 0.17 and 0.34 nm; however, the model deviates slightly at the point where the interfacial densities begin to plateau for σ_ww_ = 0.51 nm.

Table 4 shows the obtained interfacial densities for each of the LJ 9-3 walls that correspond to specific materials and the values predicted by the fit to eq 7. The predicted value slightly under-represents the LJ 9-3 wall interfacial density for heptane and tridecane, however, it does provide a reasonable estimate for all three materials.

**Table 4:** Interfacial Densities, ρ_int_, Obtained for the Specific Materials for Various Interface Types and Predicted by eq 7 in g/cm^3^

material	atomistic interface	LJ 9-3 wall	predicted value
heptane		0.52 ± 0.03	0.45
tridecane	0.57 ± 0.04	0.58 ± 0.03	0.52
graphite		0.64 ± 0.01	0.63

Although the parameters obtained from this fitting will only apply to glycine solution films of this specific thickness and concentration, the accuracy of this simple model demonstrates that the strength of the dispersion interactions has a consistent and predictable effect on the solution composition at the interface. This will allow the effect of dispersion interactions on the interfacial composition of glycine solutions to be estimated using only the LJ parameterization process at a significantly reduced computational cost compared to a full MD simulation of a solution film.

It would be expected that for higher wall strengths ρ_int_ will reach a plateau as the interface becomes saturated with glycine molecules. Figure 9 shows such a plateau for ϵ_ww_ values above 10 kcal/mol; however, from the density profiles shown in Figure 8, it can be seen that there is a clear depletion of glycine molecules within the centers of the films. This suggests that the plateau in ρ_int_ observed here may be artificially lowered by the small film size used in these simulations. In real solutions, where there is an effectively infinite reservoir of glycine molecules, the interfacial concentration may be greater than is found here. Finite size effects will be explored in Section 3.3.

The orientation of molecules in the interfacial region has been analyzed by calculating the bond orientation parameter, *P*_2_, as a function of distance from the surface. The *P*_2_ profiles show similar behavior across all wall parameters as is seen in the *P*_2_ profile shown in Figure 6, with the C–C bond tending to orient parallel to the surface. A similar, albeit weaker, orientation of the glycine molecules can be observed within the second interfacial layer that forms at higher wall strengths. The full set of *P*_2_ profiles are provided in the Supporting Information.

This behavior of glycine near the interface is similar to the previously observed behavior of physical adsorption of glycine on graphite through evaporation from ethanol,^33^ where it was found that multiple layers of glycine formed initially on the surface, but over the course of time two highly ordered layers of glycine formed. This multilayer adsorption of glycine shows interesting parallels to the concentration enhancement of glycine in aqueous solutions at the graphite interface simulated here; however, it is important to note that the ordering of the glycine molecules within the double layer formed experimentally was with the C–C bond oriented perpendicular to the surface in contrast to the parallel orientation observed here.

**Figure 10:**
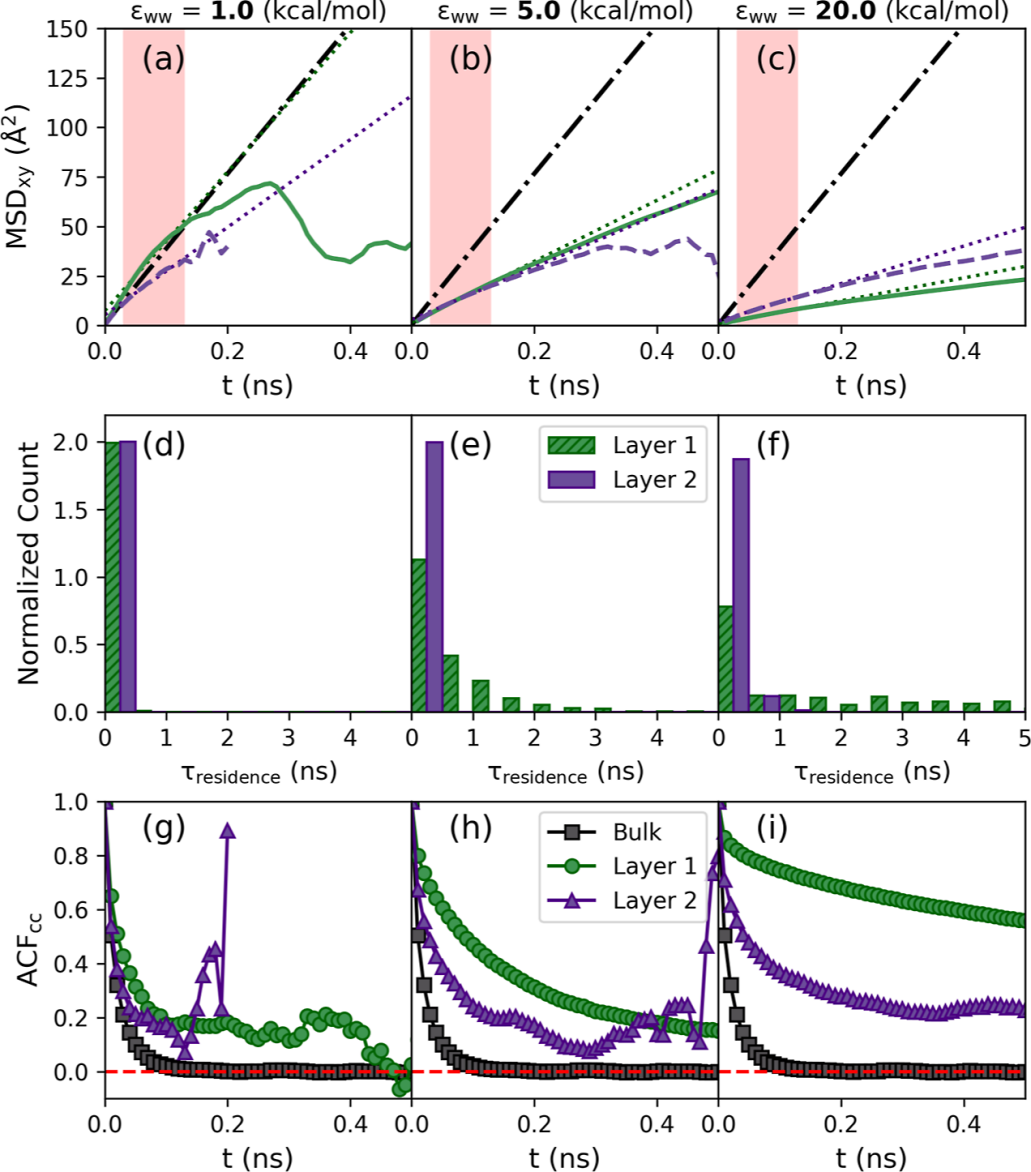
Top row shows MSD_*xy*_ of the first layer (solid green line), second layer (dashed purple line), and bulk solution (black dash-dotted line). The red area indicates the region used for the linear fit (dotted lines) used to obtain the diffusion coefficients. The second row shows the residence times of glycine molecules in the first and second layers. The third row shows the ACF of the C–C bond orientation in the first and second layers compared to bulk solution. Graphs from left to right correspond to ϵ_ww_ = 1.0, 10.0, and 20.0 kcal/mol, respectively. All graphs are for σ_ww_ = 0.34 nm.

The translational and rotational mobility of the glycine molecules within the first two interfacial layers was investigated by once again examining MSD_*xy*_, ACF_cc_, and τ_residence_. The results for LJ 9-3 walls with low (ϵ_ww_ = 1.0 kcal/mol), medium (ϵ_ww_ = 5.0 kcal/mol), and high (ϵ_ww_ = 20.0 kcal/mol) interaction strengths are presented in Figure 10.

All three metrics show that the dynamics of the glycine molecules at the interface are strongly influenced by ϵ_ww_. This is consistent with the effects observed for the composition of the solution at the interface, as the dynamics would be expected to decrease with increasing concentration. The σ_ww_ parameter is 0.34 nm in all three cases, as only a weak effect is observed for varying σ_ww_. We note that there is significant noise in the data for the wall with the weak interaction strength due to the low residence times of glycine molecules at this interface.

For lower wall strengths, where the density of the first layer is lower than that of the second, the MSD shows that molecules within the first layer have greater parallel mobility than those within the second layer. As the wall strength increases, and the interfacial region becomes more concentrated, we see the mobility of the molecules within interfacial layers decreasing, as would be expected for a region of higher concentration.^32^

Analysis of the residence times, shown in Figure 10d–f, of the glycine molecules within the interfacial layers provide insights into the mobility of the molecules perpendicular to the surface. We see that the molecules remain very mobile in the perpendicular direction within the second layer, with only a slight reduction in the mobility at the higher wall strengths (ϵ_ww_ ≥ 10 kcal/mol). Within the first layer, we see a greater reduction in the perpendicular mobility, however, we note that even at the higher wall strengths the molecules are reasonably mobile, with a majority of molecules having a residence time of 0.5 ns or less.

Finally, we examined the rotational mobility of the glycine molecules using the ACF of the C–C bond orientation of the glycine molecules, as shown in Figure 10g–i. In this case, we see that across the full range of wall parameters there is a reduction in the rotational mobility of the glycine molecules when compared to bulk solution. For ϵ_ww_ = 1.0 kcal/mol, the short residence times of the glycine molecules within the interfacial layers make it challenging to compare the ACF with the bulk ACF, but the limited data does suggest that even at the weakest wall strengths the rotational mobility is reduced. This is an expected result when the ordered nature of the interfacial layers, revealed by the *P*_2_ profiles, is taken into consideration.

The full set of dynamic properties for all wall strengths is included in the Supporting Information, alongside further quantitative analysis of the mobility of the glycine molecules within the interfacial layers, including the diffusion coefficients and decay times as described in Section 2.4.

### 3.3 Effect of System Size

As discussed in the previous section, the results of these simulations are influenced by finite size effects due to the depletion of glycine in the rest of the film. It can be expected that the interfacial concentration enhancement is hindered by this depletion and that if more glycine molecules were available within the system there may be an even greater enhancement of concentration at the interface. To test the finite size effects, we took two approaches to increase the number of glycine molecules available: (1) the overall concentration of the glycine solution was increased and (2) the thickness of the glycine solution films was increased. The graphite LJ 9-3 wall was selected as a test system, as of the three materials modeled, graphite is predicted to have the highest interfacial interactions and therefore exhibits the most severe depletion of glycine in the center of the film. Films of four different thicknesses at concentrations of 296.7 and 500.7 g/kg (all films are listed in the Supporting Information) were simulated in contact with the graphite LJ 9-3 wall to determine the effects of the system size on the interfacial solution composition.

**Figure 11:**
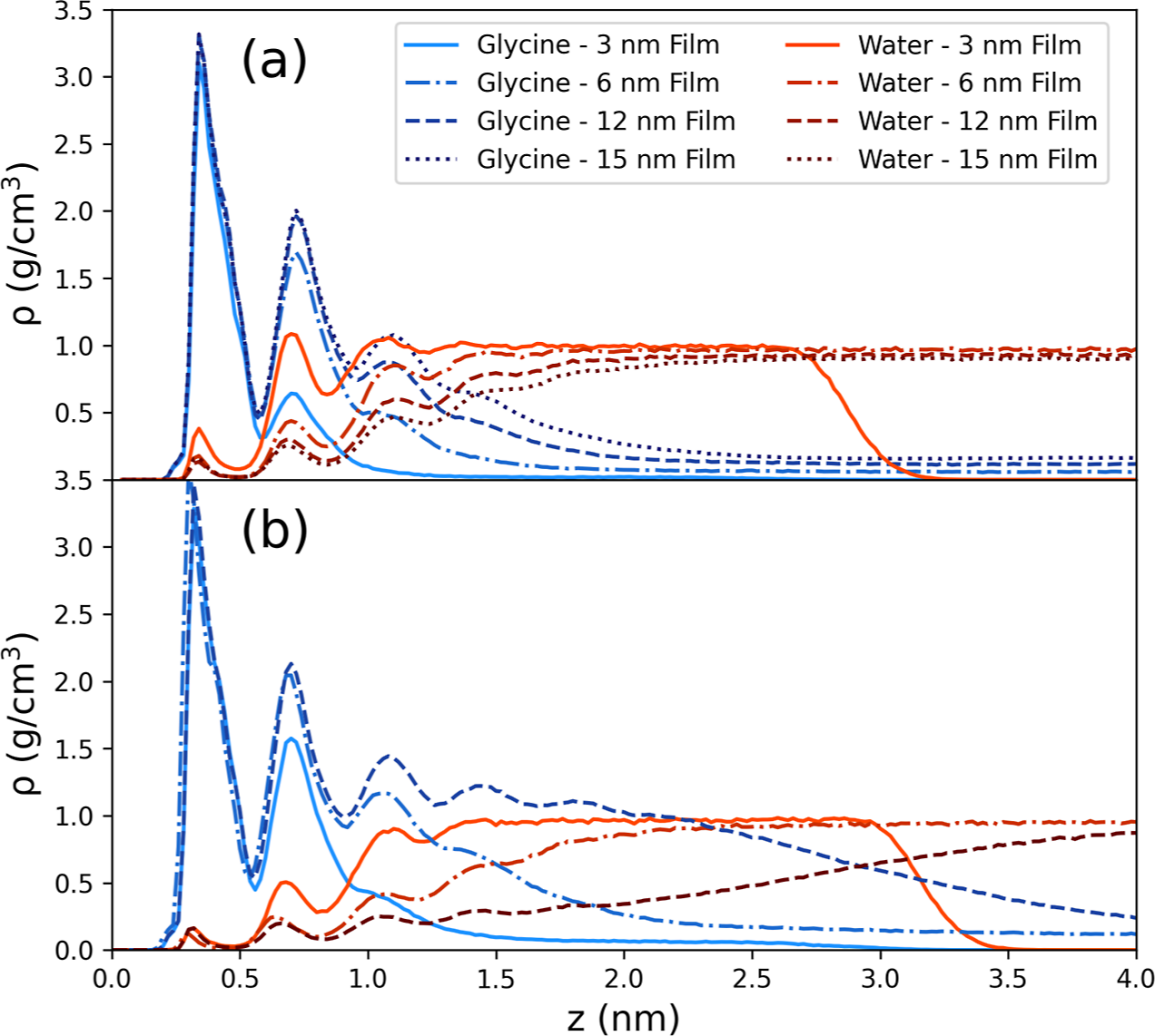
Density profiles for glycine and water for solution films of thickness 3–15 nm at concentrations of (a) 296.7 and (b) 500.7 g/kg in contact with the LJ 9-3 walls parameterized to represent graphite.

The density profiles for each of these simulations are presented in Figure 11 and show that, as expected, increasing the total amount of glycine available results in a higher density of glycine at the interface. The first glycine density peak is relatively stable, with only a modest increase as the system size increases, which is likely due to saturation of the first layer. For the thinnest film, increasing the concentration results in a significant increase in the second peak and the formation of additional peaks, particularly in the water profile, are apparent. However, for the thicker films, the second glycine peak is similar for both concentrations. As the film thickness increases, additional peaks begin to form. In the high concentration 12 nm film, the dense glycine region extends far beyond the 1 nm interfacial region that was previously defined. This extended dense region does not appear to be as structured as the well-defined peaks near the interface. The *P*_2_ profile, shown in the Supporting Information, reveals that there is no significant ordering within this dense region, beyond the initial four peaks, and there is only a slight tendency for the molecules to arrange parallel with the surface. This is a dense and disordered region of concentrated glycine that may be similar to pre-nucleation clusters in non-classical nucleation mechanisms.^6,7,34^

**Figure 12:**
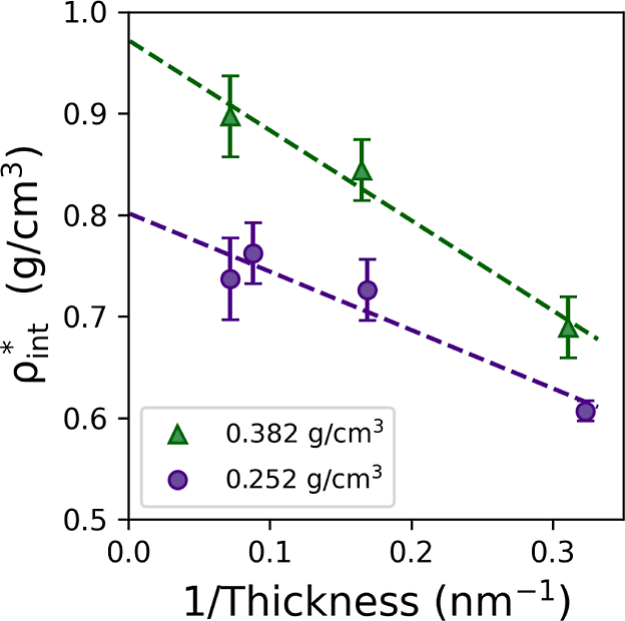
Interfacial glycine densities obtained for glycine solution films of varying thickness, with concentrations that result in bulk glycine densities of 0.252 and 0.382 g/cm^3^ in homogeneous solutions, in contact with the LJ 9-3 wall representing graphite plotted against the inverse of the solution film thickness. Extrapolating back to zero will represent a solution film of infinite thickness which is representative of a true bulk solution. Bulk solutions with these glycine densities correspond to concentrations of (a) 296.7 and (b) 500.7 g/kg.

From the density profiles shown in Figure 11, it is clear that the defined interfacial region of 1 nm is no longer suitable for quantifying the effects of the interface on the solution composition. It is therefore necessary to find a method to consistently define the interfacial region for films of different sizes. It would be expected that as you move away from the interfacial region into the center of the film the density will reach a constant value as it reaches a bulk region. The derivative of the density profile will reach a value of zero when the density reaches a constant value and can therefore be used to determine the interfacial region, as described in the Supporting Information. This process was used for each of the films simulated in contact with the LJ 9-3 wall representing graphite and the resulting interfacial glycine densities, denoted as ρ_int_^*^, for each film as shown in Figure 12. As expected, the interfacial density increases with both the film thickness and concentration, demonstrating that depletion of the solution is limiting the concentration enhancement effect observed within the simulations.

In Figure 12, the interfacial densities are plotted against the inverse of solution film thickness, which enables extrapolation back to zero, corresponding to a film of infinite thickness. A linear fit predicts interfacial densities of 0.81 and 0.97 g/cm^3^ for films of infinite thickness at concentrations of 296.7 and 500.7 g/kg, respectively. This gives an estimate for the interfacial density that would be expected for the bulk solution in contact with the interface. In comparison, the average densities of bulk solutions (or the bulk region of films of infinite thickness) would be 0.252 and 0.382 g/cm^3^, respectively. This shows that a 2–3 fold increase in the density of glycine in the interfacial region compared to that in the bulk solution can be expected for non-polar interfaces in contact with glycine solutions.

### 3.4 Effect of Electrostatics

Thus far, we have only examined the effects of dispersion interactions between the surface and the solution. In this section, the impacts of electrostatics on the solution composition at the interface will be explored. This has been investigated through simulations of a glycine solution film in contact with atomistic surfaces with various combinations of LJ parameters and atomic charge sets.

In this section, we use fluoroalkanes and alkanes to explore electrostatic effects on solution composition. PTFE has a similar molecular structure to alkanes, with all hydrogen atoms replaced with fluorine atoms. The high electronegativity of fluorine results in much larger partial charges in PTFE than alkanes. In addition, PTFE has previously been shown to increase the nucleation rate of glycine from aqueous solutions.^12^ These attributes made PTFE an attractive choice for this test. We have represented PTFE using the same atomic configuration as for tridecane, replacing the hydrogen atoms with fluorines to create perfluorotridecane. We have simulated glycine solutions in contact with the perfluorotridecane using the same crystal structure used in the simulations of tridecane presented in Section 2.4. By using the same atomic structure for the two interfaces, we ensure that the atomic density is equal between the two interfacial systems, and the surface–solution interactions can be changed from tridecane to perfluorotridecane simply by adjusting the intermolecular potentials.

It is important to note that alongside the difference in electrostatic charges between the PTFE and tridecane molecules, there are also differences in the LJ interactions for the two materials. For example, the fluorine atoms within PTFE have ϵ_ii_ and σ_ii_ parameters of 0.061 kcal/mol and 0.312 nm, respectively, in contrast to the ϵ_ii_ and σ_ii_ parameters of 0.0124 kcal/mol and 0.266 nm for the hydrogens in tridecane (the full set of intermolecular parameters are provided in the Supporting Information). It is therefore necessary to decouple the effects of the electrostatic interactions and the dispersion interactions on the solution composition at the interface. This has been achieved by separating the LJ parameters and electrostatic charges into separate parameter sets that can be applied to the surface. Two LJ parameter sets have been considered, PTFE and tridecane, and three charge sets: tridecane, PTFE, and 2× PTFE. The third charge set (2× PTFE) is artificially large to strengthen the effect of the electrostatics, independently of dispersion interactions.

**Figure 13:**
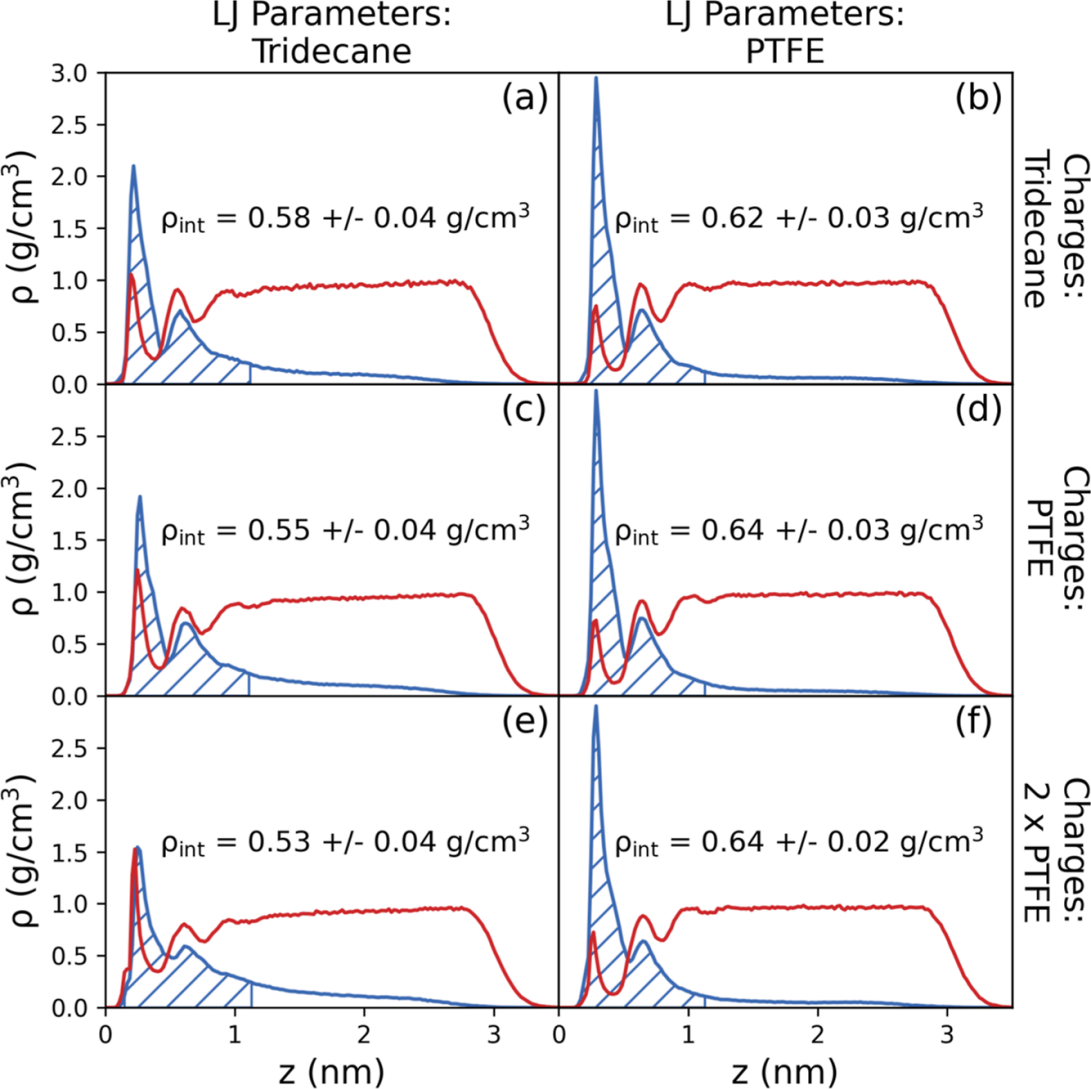
Density profiles for the tridecane/PTFE interface simulations. The left and right columns show the simulations with the LJ parameters for tridecane and PTFE, respectively. The top, middle, and bottom rows show the simulations with tridecane, PTFE, and 2× PTFE charges, respectively.

Figure 13 presents the density profiles of the 296.7 g/kg, 3.1 nm film simulated using the six possible combinations of the LJ and electrostatic parameter sets. Simulations with tridecane and perfluorotridecane LJ parameters are shown on the left and right, respectively. The first, second, and third row show the results with tridecane, PTFE, and 2× PTFE charges, respectively.

As expected, the weaker tridecane LJ parameters give a lower interfacial glycine density than the stronger PTFE LJ parameters, for all charge sets. For the tridecane LJ parameters, it can be seen that as the charges increase (Figure 13a,c,e) the interfacial density of glycine slightly decreases, although the average values fall within the errors. However, it can be seen that the peak height of the first interfacial peak decreases by around 25%. In contrast, for the PTFE LJ parameter simulations there is a small, but statistically insignificant, increase. It is possible, however, that for weak dispersion interactions, electrostatics may become more important.

For these systems, the effect of electrostatic interactions appears to be relatively weak compared to dispersion interactions, which strongly affect the interfacial concentration of glycine in aqueous solutions adjacent to the hydrophobic interface corresponding to PTFE.

### 3.5 Impact of Interfacial Concentration Enhancement on Heterogeneous Nucleation

Based on the simulation results presented here, it can be seen that non-polar materials in contact with aqueous glycine solutions result in a distinct interfacial region where glycine concentration will be much higher than in the bulk solution, with a significantly lower mobility and with preferential orientation in the first glycine layer adjacent to the surface. This can be expected to enhance heterogeneous nucleation of glycine beyond what is expected from classical nucleation theory. Although the free energy (or chemical potential) of glycine in the interfacial region would be the same as in the bulk solution, local density and structure in the interfacial region would be different and therefore different pathways would be available in the free-energy landscape so that non-classical nucleation mechanisms could become accessible with lower energy barriers compared to those available in the bulk solution.

This is consistent with previous observation where glycine nucleation was enhanced by the presence of non-polar interfaces, including PTFE,^12^ tridecane,^13^ and graphene. Boyes et al. investigated the effect of graphene as a substrate on the nucleation behavior of glycine in small water droplets and reported a reduction in induction times in their Supporting Information,^35^ which corresponds to an increase in the nucleation rate at a given supersaturation.^36^

It is clear that the strength of the dispersion interactions between the surface and the solution significantly impacts the solution composition within the interfacial region. This suggests that during heterogeneous nucleation the bulk solution is not necessarily representative of the environment in which nucleation is occurring. This is a key insight when comparing nucleation kinetics in different systems as experiments with the same overall concentration may have significantly different interfacial concentrations and solution structures. This means that the heterogeneous nucleation rates may vary significantly for different experimental setups that have different interfaces present, such as oils in microfluidic experiments, glass vials, or polymer microwells, tubings, and stirrers.

## 4 Conclusions

In this work, we investigated the effects of surface–solution interactions on the solution composition, molecular orientation, and dynamics within the interfacial region of aqueous glycine solution adjacent to non-polar, hydrophobic surfaces. Simulations reveal that the dispersion interactions between the surface and the solution have a major effect on the concentration of glycine at the interface, with the interfacial density of glycine increasing significantly (up to 2–3 fold) with the strength of the surface–solution interactions. This is resulting from a competition between solute and solvent molecules to be or not to be near the interface.

We developed a novel parameterization process that allows for the LJ 9-3 potential to be mapped onto real materials. This parameterization process has been validated using the tridecane–solution interface, where we have demonstrated that the LJ 9-3 wall is capable of reproducing the solution composition and molecular orientation with excellent agreement, and dynamic properties with good agreement, at the interface with a significantly reduced computational cost compared to a fully atomistic interface. This procedure was also applied to heptane and graphite in order to generate suitable LJ 9-3 walls to represent each of these materials.

An empirical fitting procedure was used to describe the variation of the interfacial glycine density with the ϵ_ww_ and σ_ww_ parameters of the LJ 9-3 walls. The model was able to reasonably estimate the interfacial density of glycine for a solution film of the same size and concentration in contact with a LJ 9-3 wall of a given set of ϵ_ww_ and σ_ww_ parameters. The combination of the parameterization process and the fitting procedure will enable the effects of interface materials on concentration enhancement in the interfacial region to be estimated using a computationally inexpensive LJ parameterization process, without the need of MD simulations. This can, therefore, be used as an effective tool for screening materials for heterogeneous nucleation enhancement or suppression.

As the simulated films are of the order of nm, we investigated finite size effects and demonstrated that the interfacial concentration enhancement is likely to be even greater in larger systems corresponding to bulk solutions compared to what is observed within the nanoscale thin films.

Finally, we investigated the influence of electrostatic interactions between the surface and the solution and contrasted this with the effects of dispersion interactions and found that the effect of the dispersion interactions on the solution composition is much greater than the effect of electrostatics for surfaces representing PTFE.

This work demonstrates that dispersion interactions between a surface and solution can have significant impacts on solution composition, structure, and dynamics within the interfacial region. These effects can be accurately reproduced using a simple model interface allowing this behavior to be captured at a significantly reduced computational cost in contrast to simulating a fully atomistic interface. Additionally, once a baseline of simulations have been performed, the effects of the interface on the solution composition can be reasonably estimated from wall parameters obtained from a computationally inexpensive parameterization process, providing opportunities for fast material screening in the future.

## Supporting Information Available

Simulation details, including force field parameters, detailed film sizes, and simulation times; snapshot of the crystalline tridecane surface; detailed surface–atom parameterization example; complete sets of results for bond orientation profiles, MSD_*xy*_, ACF_cc_ and interfacial residence times for the grid of LJ 9-3 simulations; more detailed analysis of the dynamic properties obtained from the grid of LJ 9-3 simulations; bond orientation profile for the 500.7 g/kg, 12 nm film in contact with the LJ 9-3 wall representing graphite; and more detailed description of the process used to determine the interfacial region for thicker films (PDF)

## Notes

Simulation input files are available from the University of Strathclyde KnowledgeBase at https://doi.org/10.15129/8d54c68e-69a6-4215-8a4c-d0141d3f2baf.
