# Supplementary material for: Tuning Interfacial Concentration Enhancement through Dispersion Interactions to Facilitate Heterogeneous Nucleation
Source: J Phys Chem C Nanomater Interfaces. 2022 Sep 16;126(38):16387–400. doi: 10.1021/acs.jpcc.2c04410 (PMC9527751; doi:10.1021/acs.jpcc.2c04410)
Supplement: Supplementary file 1 — jp2c04410_si_001.pdf [file jp2c04410_si_001.pdf]

# Supporting Information for Tuning Interfacial Concentration Enhancement through Dispersion Interactions to Facilitate Heterogeneous Nucleation

David McKechnie[1,2], Paul A. Mulheran[1], Jan Sefcik[1,3], and Karen Johnston[1]

[1]Department of Chemical and Process Engineering, University of Strathclyde, Glasgow G1 1XJ, United Kingdom

[2]Doctoral Training Centre in Continuous Manufacturing and Advanced Crystallisation, University of Strathclyde, Glasgow G1 1RD, U.K.

[3]EPSRC Future Manufacturing Research Hub in Continuous Manufacturing and Advanced Crystallisation, University of Strathclyde, Glasgow G1 1RD, U.K.

August 17, 2022

## S1 Simulation Details

### S1.1 Force Field Details

Glycine molecules were modelled using GAFF [1] alongside the CNDO charge set [2]. The intramolecular and LJ parameters for glycine are given in Table S1. Figure S1 demonstrates the atom types that were selected for the glycine molecule and labelled using the GAFF naming convention. Electrostatic interactions were described as Coulomb interactions between point charges. The two oxygen (o) atoms have charges of -0.526 and -0.483, the carboxylic acid group carbon (c) has charge +0.374, the aliphatic carbon (c3) has charge +0.374, aliphatic hydrogens (hx) have charges +0.033 and +0.030, the nitrogen (n4) has charge +0.022 and the amine hydrogens (hn) have charges +0.164, +0.208 and +0.199.

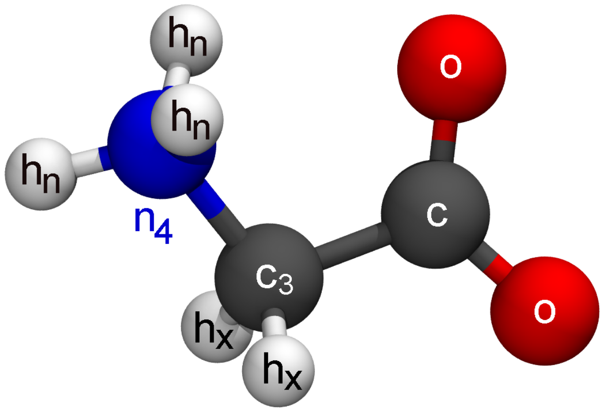


**Figure S1:** Diagram of a glycine molecule with each atom labeled with the GAFF atom type used within the simulations.

**Table S1:** GAFF force field parameters for glycine. Atom type X in the dihedral parameters denotes any atom type.

**LJ Parameters**

| **Atom Type** | $\epsilon$ (kcal/mol) | $\sigma$ ($\AA$) |
|---|---|---|
| n4 | 0.1700 | 3.25 |
| c3 | 0.1094 | 3.40 |
| c | 0.0860 | 3.40 |
| o | 0.2100 | 2.96 |
| hn | 0.0157 | 1.07 |
| hx | 0.0157 | 1.96 |

**Bond Parameters**

| **Atom Type** 1 | 2 | $\mathbf{k_b}$ (kcal/mol) | $\mathbf{r_0}$ (Å) |
|---|---|---|---|
| hn | n4 | 369 | 1.066 |
| n4 | c3 | 293.6 | 1.499 |
| c3 | hx | 338.7 | 1.091 |
| c3 | c | 328.3 | 1.508 |
| c | o | 648.0 | 1.214 |

**Angle Parameters**

| **Atom Type** 1 | 2 | 3 | $\mathbf{k_a}$ (kcal/mol) | $\theta_0$ (°) |
|---|---|---|---|---|
| hn | n4 | hn | 40.5 | 108.11 |
| hn | n4 | c3 | 46.2 | 110.11 |
| n4 | c3 | hx | 49.0 | 107.91 |
| n4 | c3 | c | 65.8 | 111.54 |
| c3 | c | o | 68.0 | 123.11 |
| o | c | o | 79.1 | 127.33 |
| hx | c3 | hx | 39.0 | 110.74 |
| c | c3 | hx | 47.4 | 108.64 |

**Dihedral Parameters**

| **Atom Type** 1 | 2 | 3 | 4 | **i** | $\mathbf{k_i}$ (kcal/mol) | $\mathbf{n_i}$ | $\mathbf{d_i}$ (o) |
|---|---|---|---|---|---|---|---|
| X | c3 | n4 | X | 1 | 0.15 | 3 | 0 |
| X | c | c3 | X | 1 | 0.00 | 2 | 180 |
| X | o | c | X | 1 | 1.10 | 2 | 180 |

Water molecules were represented using the SPC/E model [3]. The bond lengths and angles were fixed using the SHAKE algorithm. Force field parameters are provided in Table S2.

**Table S2:** Force field parameters for the SPC/E water model.

| **Parameter** | **Value** |
|---|---|
| $r_{OH}$ | 1.00 Å |
| $<_{HOH}$ | 109.47 ° |
| $\epsilon_{OO}$ | 0.1555 kcal/mol |
| $\sigma_{OO}$ | 3.166 Å |
| $q_O$ | -0.8476 |
| $q_H$ | 0.4238 |

Tridecane molecules were represented using the AMBER-ii [4] force field, which is an extension of the AMBER force field to provide a better description of long chain alkanes. This force field uses the same functional form as GAFF and the force field parameters are provided in Table S3. A charge of -0.012 is assigned to the $CH_3$ carbons and +0.012 is assigned to the connected $CH_2$ carbon. All other tridecane atoms are chargeless.

Graphite and PTFE were modelled using GAFF. As the graphite and PTFE atoms were fixed in place and no dynamics were performed, only the intermolecular interactions were required. LJ parameters are provided in Table S4. The carbon atoms of graphite were chargeless, whilst the charges for the PTFE were obtained using the AM1-BCC method within Antechamber [5] and are given in Table S5.

**Table S3:** AMBER-ii force field parameters for tridecane. Note that the dihedral term for the carbon backbone of tridecane has three combined terms, whilst all other dihedrals have one.

| | | | | | | | |
|---|---|---|---|---|---|---|---|
| **LJ Parameters** | | | | | | | |
| **Atom Type** | | | | | $\epsilon$ (kcal/mol) | | $\sigma$ (Å) |
| C (CH2) | | | | | 0.1560 | | 3.12 |
| C (CH3) | | | | | 0.1840 | | 3.12 |
| H | | | | | 0.0124 | | 2.66 |
| **Bond Parameters** | | | | | | | |
| **Atom Type** | | | | | $\mathbf{k_b}$ (kcal/mol) | | $\mathbf{r_0}$ (Å) |
| C | C | | | | 240.0 | | 1.52 |
| C (CH2) | H | | | | 340.0 | | 1.096 |
| C (CH3) | H | | | | 340.0 | | 1.092 |
| **Angle Parameters** | | | | | | | |
| **Atom Type** | | | | | $\mathbf{k_a}$ (kcal/mol) | | $\boldsymbol{\theta_0}$ (°) |
| H | C | H | | | 33.0 | | 107.0 |
| C | C | H | | | 52.0 | | 110.7 |
| C | C | C | | | 30.0 | | 109.5 |
| **Dihedral Parameters** | | | | | | | |
| **Atom Type** | | | | **i** | $\mathbf{k_i}$ (kcal/mol) | $\mathbf{n_i}$ | $\mathbf{d_i}$ (°) |
| H | C | C | C | 1 | 0.16 | 3 | 0 |
| H | C | C | H | 1 | 0.16 | 3 | 0 |
| C | C | C | C | 1 | 0.16 | 3 | 0 |
| C | C | C | C | 2 | 0.09 | 2 | 180 |
| C | C | C | C | 3 | 0.06 | 1 | 180 |

**Table S4:** GAFF LJ parameters for graphite and PTFE.

| **Atom Type** | $\epsilon$ (kcal/mol) | $\sigma$ (Å) |
|---|---|---|
| **Graphite** | | |
| ca | 0.0860 | 3.4 |
| **PTFE** | | |
| c3 | 0.1094 | 3.40 |
| f | 0.061 | 3.12 |

**Table S5:** Charges assigned to the atoms within the PTFE molecules using the AM1-BCC method.

| **Backbone Position** | $\mathbf{q_C}$ | $\mathbf{q_F}$ |
|---|---|---|
| 1 | 0.6100 | -0.2008 |
| 2 | 0.3477 | -0.1762 |
| 3 | 0.3497 | -0.1767 |
| 4 | 0.3517 | -0.1762 |
| 5 | 0.3517 | -0.1757 |
| 6 | 0.3517 | -0.1757 |
| 7 | 0.3517 | -0.1757 |
| 8 | 0.3517 | -0.1757 |
| 9 | 0.3517 | -0.1757 |
| 10 | 0.3517 | -0.1757 |
| 11 | 0.3497 | -0.1767 |
| 12 | 0.3477 | -0.1762 |
| 13 | 0.6100 | -0.2008 |

## S1.2 System Details

Table S6 shows the system information and simulation times for the glycine solution films used in the MD simulations.

**Table S6:** System details and simulation times for each of the glycine solution films.

| **Concentration** (g/kg) | $\mathbf{N}_{\text{glycine}}$ | $\mathbf{N}_{\text{water}}$ | **Film Thickness** (nm) | $t_{\text{equil}}$ (ns) | $t_{\text{prod}}$ (ns) |
|---|---|---|---|---|---|
| 296.7 | 64 | 900 | 3.1 | 25 | 15 |
| 296.7 | 128 | 1800 | 5.9 | 35 | 25 |
| 296.7 | 256 | 3600 | 11.4 | 40 | 40 |
| 296.7 | 320 | 4500 | 13.9 | 60 | 70 |
| 500.7 | 108 | 900 | 3.2 | 25 | 15 |
| 500.7 | 216 | 1800 | 6.1 | 35 | 25 |
| 500.7 | 432 | 3600 | 12.2 | 40 | 40 |

The simulation of the bulk solution was performed using the 5.9 nm thick, 296.7 g/kg film, simulated with fully periodic boundaries for 10 ns in the NPT ensemble.

## S2 Snapshot of the Tridecane Crystalline Interface

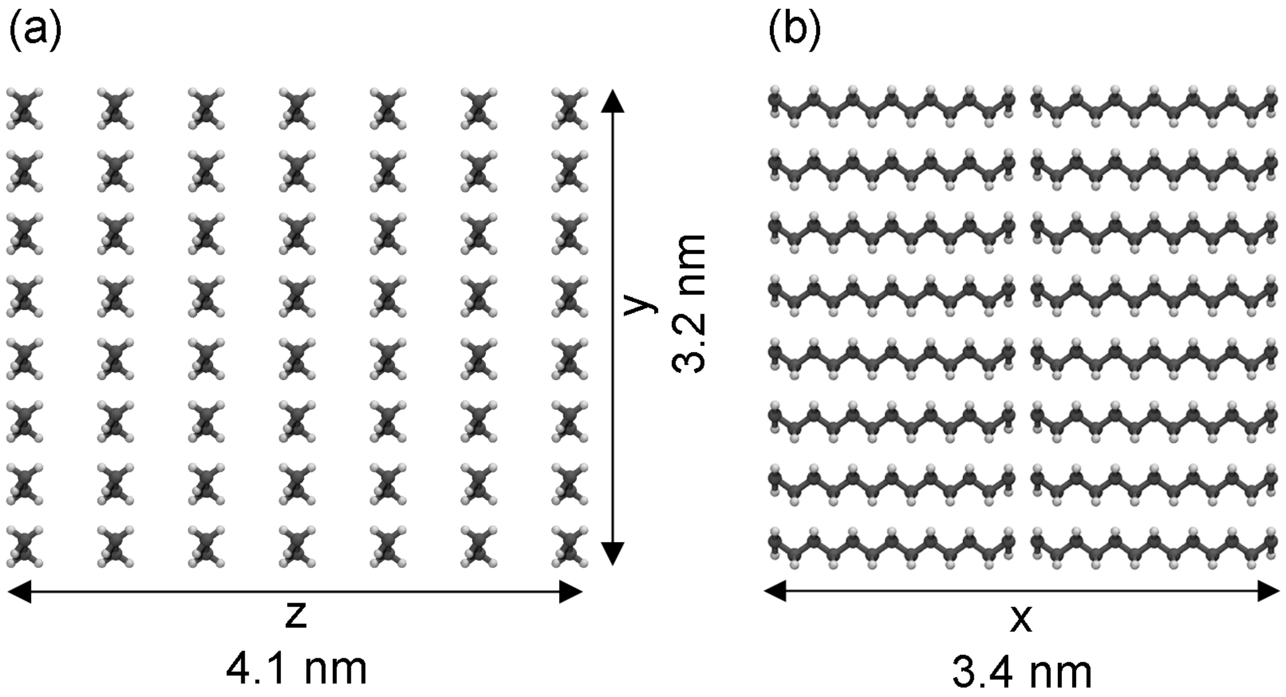


**Figure S2:** Snapshot of the crystalline tridecane slab. (a) shows the z-y plane while (b) shows the x-y plane. Carbon atoms are shwon in grey, while hydrogen atoms are shown in qhite. The dimensions of the slab are provided in each direction.

## S3 Interface–Atom Parameterisation Example: Tridecane and Nitrogen

In order to provide extra clarity on the LJ parameterisation process, this section will describe how the parameters $\epsilon_{iw}$ and $\sigma_{iw}$ were obtained for the interaction between the LJ 9-3 wall representing tridecane and the nitrogen atom within the glycine molecules.

The nitrogen atom within the glycine molecule, n4, has LJ parameters of $\epsilon_{ii} = 0.17$ kcal/mol and $\sigma_{ii} = 0.325$ nm. 36 evenly spaced positions were selected in the $x$-$y$ plane of the atomistic, crystalline tridecane slab. A nitrogen atom was placed at a distance, $z$, of 0.01 nm above the interface at the first $x$-$y$ position, and the total LJ 12-6 interaction between the tridecane slab and the nitrogen atom was calculated. The distance was then increased in 0.01 nm steps, up to a maximum of $z = 1.4$ nm, calculating the LJ 12-6 interaction at each step. Figure S3 (a) shows a snapshot of the nitrogen interacting with the tridecane slab.

This provides a $z$-dependent potential between the atom and the interface, such as the one shown in Figure S3 (b). Equation 1 is then fit to this potential to obtain $\epsilon_{iw}$ and $\sigma_{iw}$ parameters that describe the interaction between the nitrogen atom and the tridecane interface at the specific $x$-$y$ position. Measurements close to the interface resulted in extreme values of $E_{LJ}$ due to repulsion between the nitrogen atom and the tridecane interface. Due to this, only values of $E_{LJ} < 3.0$ kcal/mol were included in the fitting of equation 1 to better capture the behaviour of the potential well. This does result in deviation from the $z$-dependent potential at $z$ values close to the interface, however this will not influence the simulation results as atoms will be unable to reach this highly repulsive region under typical conditions. Fitting equation 1 to the data shown in Figure S3 (b) resulted in parameters $\epsilon_{iw} = 1.19$ kcal/mol and $\sigma_{iw} = 0.301$ nm.

This process was repeated for each of the 36 $x$-$y$ positions across the face of the tridecane slab, and each of the $z$-dependent potentials are shown in Figure S3 (c) as the coloured data sets. The average $\epsilon_{iw}$ and $\sigma_{iw}$ values are 1.22 kcal/mol and 0.316 nm respectively, and the resulting LJ 9-3 potential is shown as the black line in Figure S3 (c). The values $\epsilon_{iw}$ and $0.715\sigma_{iw}$ are used in the fitting of equations 3 and 2 in order to obtain the wall parameters, $\epsilon_{ww}$ and $\sigma_{ww}$, to represent tridecane. These values correspond to the red squares plotted at $\epsilon_{ii} = 0.17$ kcal/mol and $\sigma_{ii} = 0.325$ nm in Figure 3.

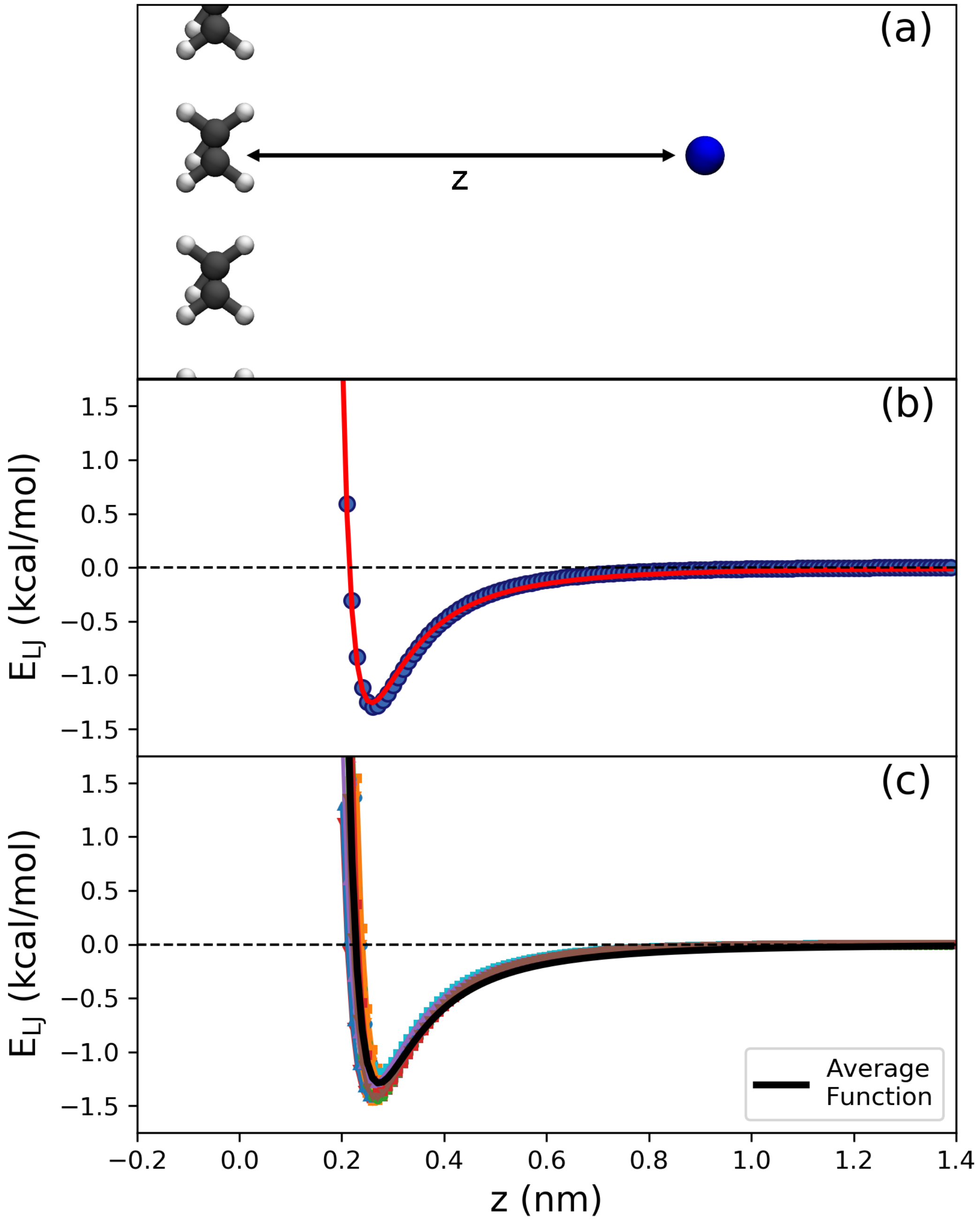


**Figure S3:** (a) Schematic to demonstrate the parameterisation process for the crystalline tridecane system. The LJ interactions between the tridecane slab and the blue nitrogen atom are calculated for the distance, $z$, from the interface. (b) The LJ interaction mapped out for a nitrogen atom interacting with the tridecane at varying distances at one $x$-$y$ position. The red line shows the fit to equation 1 to obtain $\epsilon_{iw}$ and $\sigma_{iw}$ (c) LJ interactions measured for a nitrogen atom interacting with the tridecane at each of the 36 $x$-$y$ positions. Each coloured set of points represents one $x$-$y$ position, while the black line shows the function using the average $\epsilon_{iw}$ and $\sigma_{iw}$ values.

## S4 Bond Orientation Profiles of Glycine Molecules at LJ 9-3 Interfaces

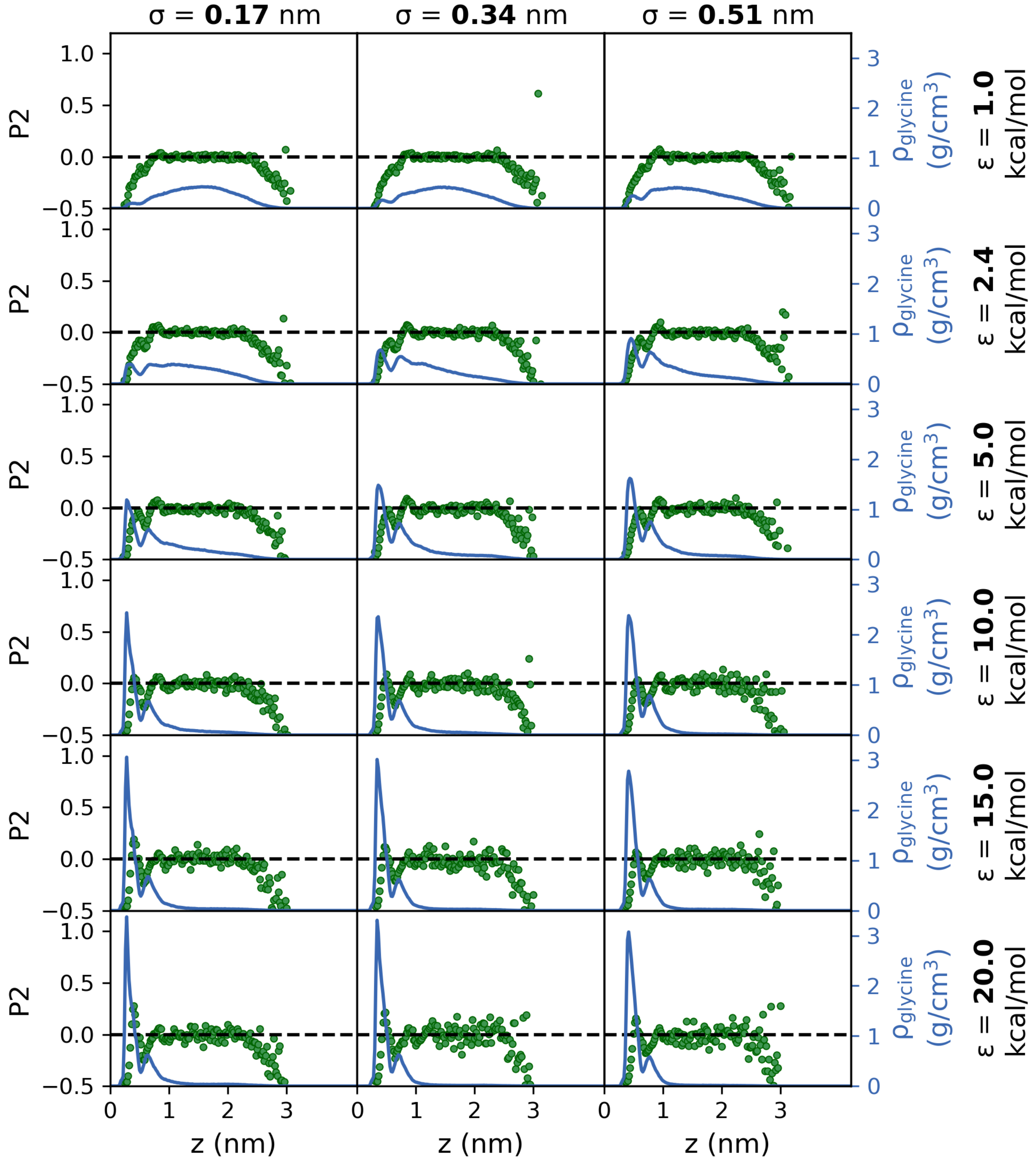


**Figure S4:** $P_2$ profiles in the $z$-direction of the C-C bond vector of glycine (green circles) for different wall interaction parameters. The density profiles of glycine are plotted (dark blue line) on the secondary axis to allow the position of the ordered regions to be determined.

## S5 Dynamic Properties of Glycine at LJ 9-3 Interfaces

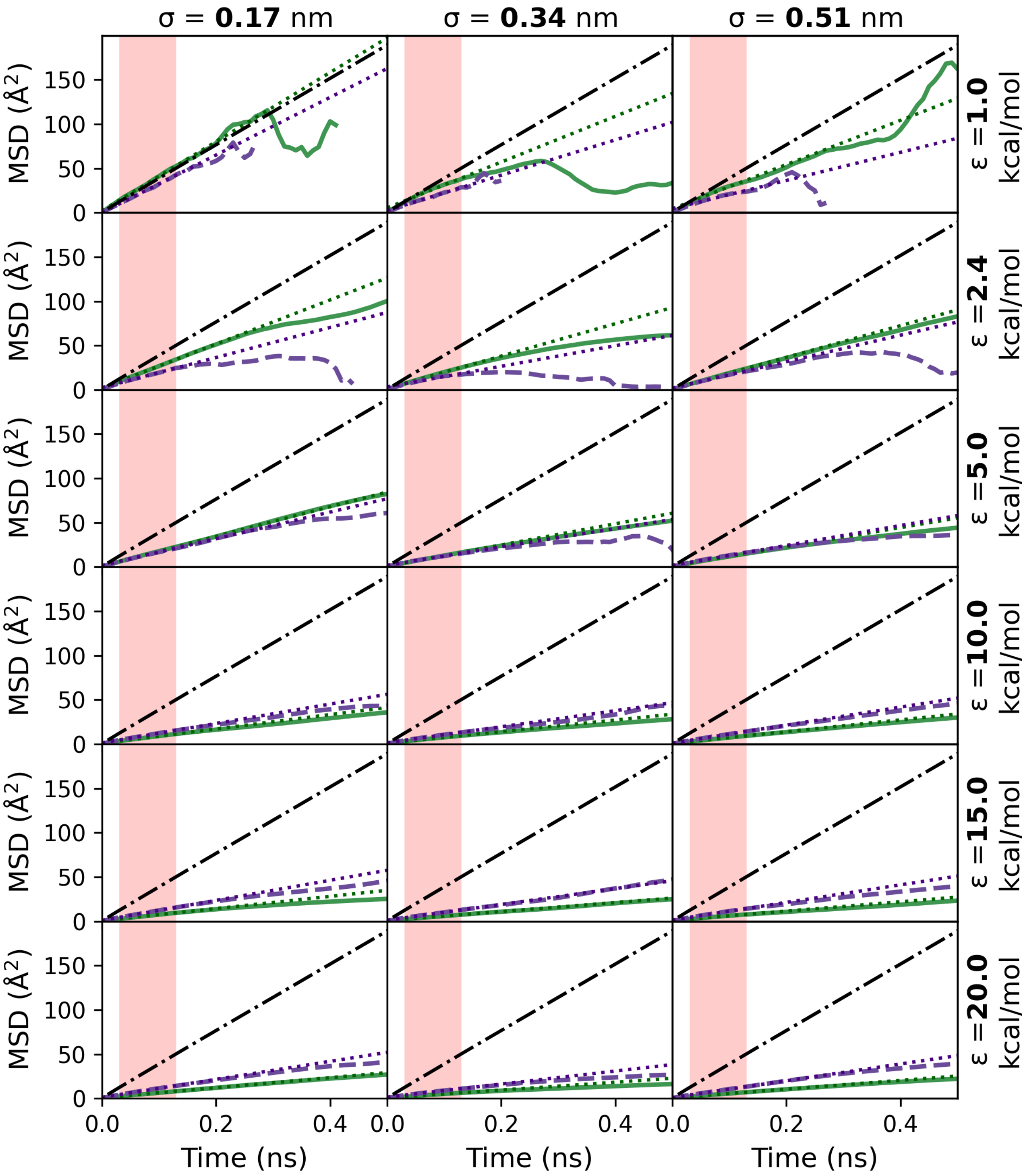


**Figure S5:** Mean squared displacement (MSDs) of glycine within the 1st (green solid line) and 2nd (purple dashed line) interfacial layers for different wall interaction parameters compared to those of bulk glycine solution (black dash-dotted line). The red shaded area indicates the region that the linear fit was applied to to determine the diffusion coefficients parallel to the interface. The dotted lines shows the linear fits to this region.

Diffusion coefficients have been determined for the parallel movement of the glycine molecules within

each of the interfacial layers and are plotted for each wall strength in Figure S6. The parallel mobility decreases as $\epsilon_{\mathrm{ww}}$ increases, although for values of $\epsilon_{\mathrm{ww}}$ above 10 kcal/mol the decrease become smaller. This is at least partly due to the concentration of the two layers not increasing significantly for the higher wall strengths as the remainder of the solution is depleted of glycine due to finite size effects. There is a slight decrease in the diffusion coefficient as $\sigma_{\mathrm{ww}}$ increases from 0.17 to 0.51 nm, however this effect is much weaker than is observed for $\epsilon_{\mathrm{ww}}$, and the diffusion coefficients within the first layer are very similar for $\sigma_{\mathrm{ww}} = 0.34$ and 0.51 nm.

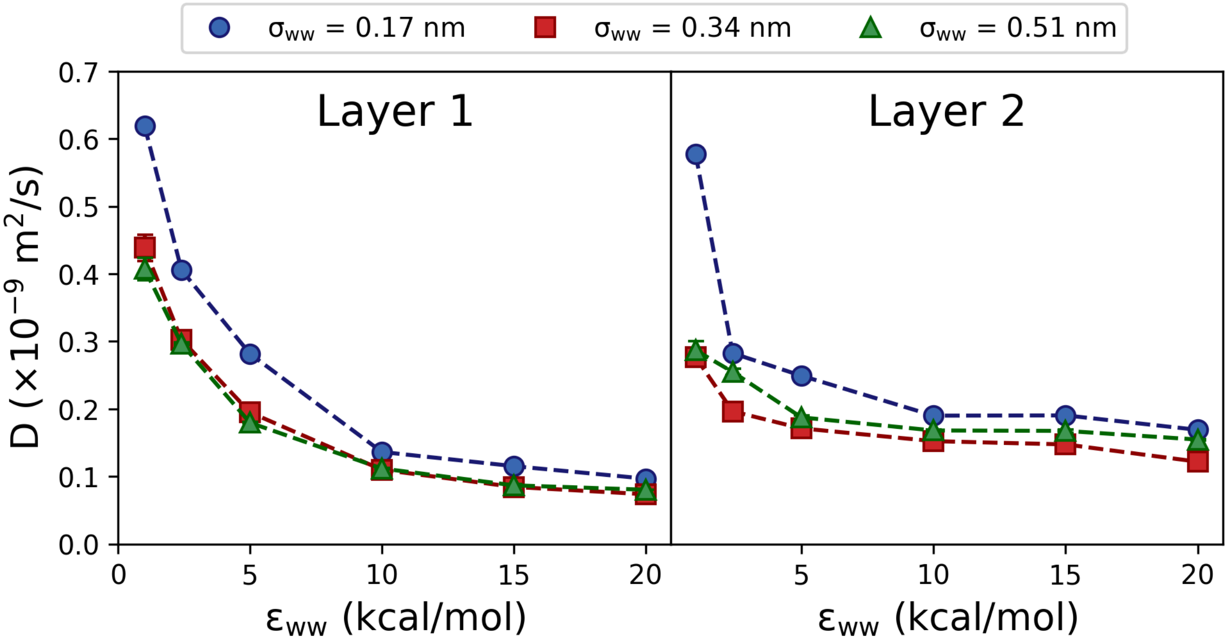


**Figure S6:** Diffusion coefficients in the x-y direction (parallel to the interface) obtained for the glycine molecules in the first (left) and second (right) interfacial layers for different wall interaction parameters.

Figure S9 shows the decay times, as described in section , for the interfacial layers at each wall strength investigated. We see similar behaviour as observed for the diffusion coefficients, with the decay time increasing with $\epsilon_{\mathrm{ww}}$ indicating a reduction in the rotational mobility of the glycine molecules. Once again, a small reduction in the mobility of the molecules is observed for increasing $\sigma_{\mathrm{ww}}$ however, this effect is much less pronounced than observed for $\epsilon_{\mathrm{ww}}$.

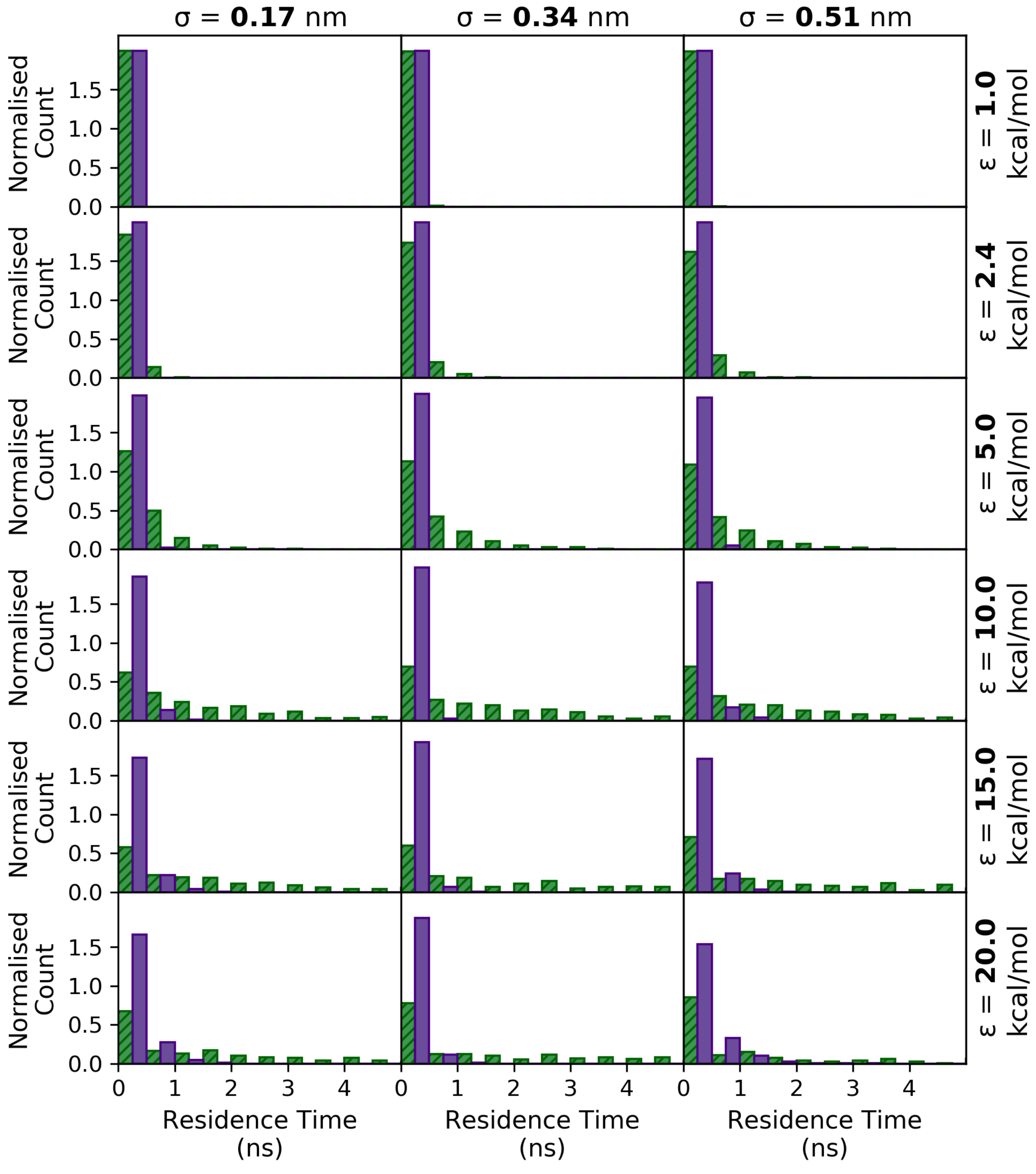


**Figure S7:** Residence times of glycine molecules within the 1st (green hatched) and 2nd (purple) interfacial layers for different wall interaction parameters.

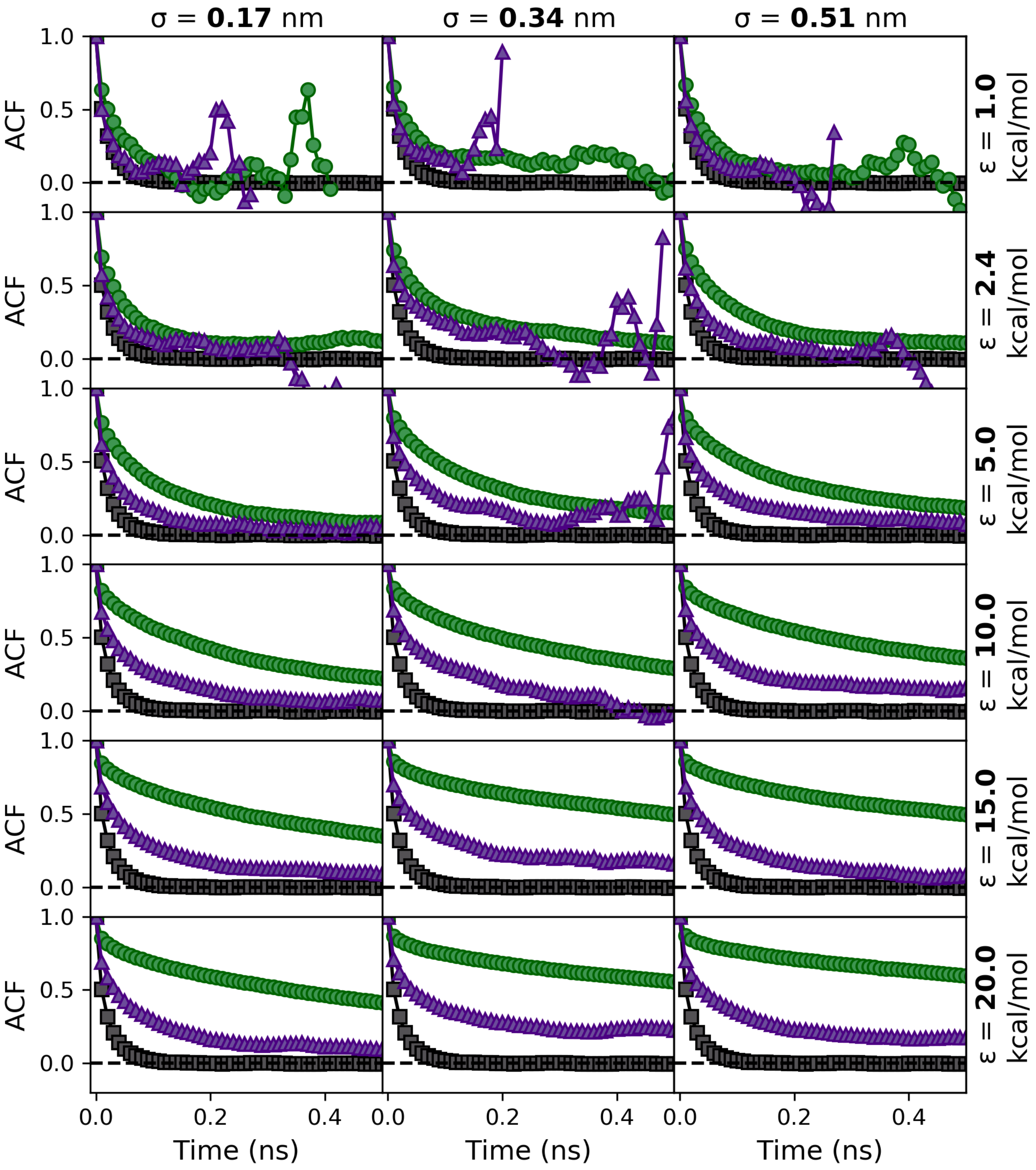


**Figure S8:** Autocorrelation functions of the C-C bond in glycine within the 1st (green circles) and 2nd (purple triangles) interfacial layers for different wall interaction parameters compared to those of bulk glycine solution (grey squares).

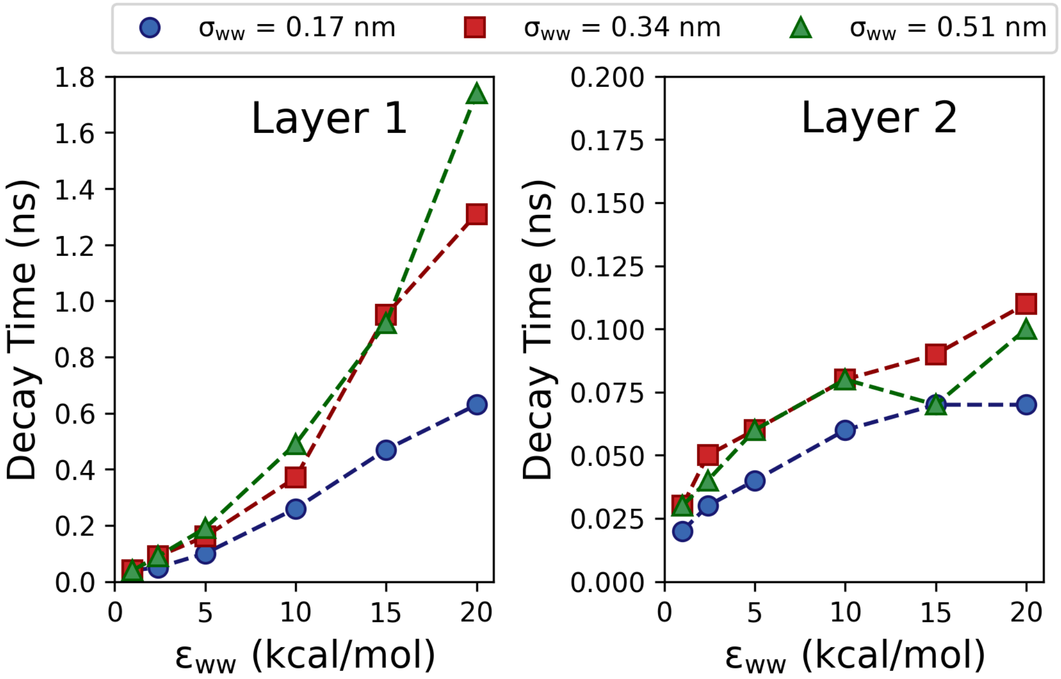


**Figure S9:** Decay times obtained for the glycine molecules in the first (left) and second (right) interfacial layers for different wall interaction parameters.

## S6 $P_2$ Profile for 500.7 g/kg, 12 nm Film

The simulation of the largest, most concentrated glycine solution film in contact with the LJ 9-3 wall representing graphite resulted in multiple dense layers of glycine at the interface followed by a tailing off region towards the bulk of the film. The $P_2$ profile, shown in Figure S10, demonstrates that there is not significant ordering of the glycine molecules within the tailing off region.

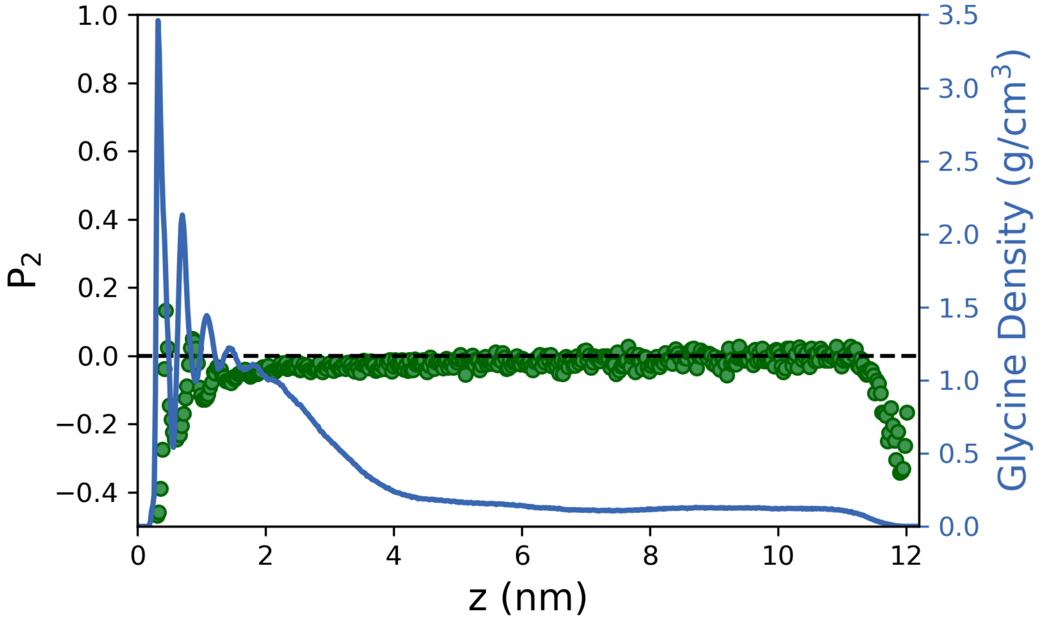


**Figure S10:** The bond order profile obtained for the 500.7 g/kg, 12 nm glycine solution film in contact with the LJ 9-3 wall representing graphite. The density profile of the glycine solution is plotted on the secondary axis.

## S7 Interfacial Region Definition

For simulations of larger and more concentrated glycine solutions, the effects of the interface extends beyond the initially defined 1 nm interfacial region. To account for this, a new method for determining a suitable interfacial region was developed in an attempt to provide a fair comparison between solution films of different sizes and concentrations. A Savitzky Golay filter [6] was used to smooth the density profile as is shown in Figure S11 (a). The filter fits a polynomial to a moving window throughout the data, and was applied using third order polynomials and a window size of five. The filter accurately captures the density profile, but it does slightly understate the peak closes to the interface. However, for determining the interfacial region it is important that the tail down to the bulk-like region is accurately captured, which the Savitzky-Golay filter achieves successfully. The derivative of this smoothed data was taken (see Figure S11 (b)), and the limit of

the interfacial region was defined at the point at which this derivative first crosses the $x$-axis, excluding the interfacial peaks, as is shown by the vertical line.

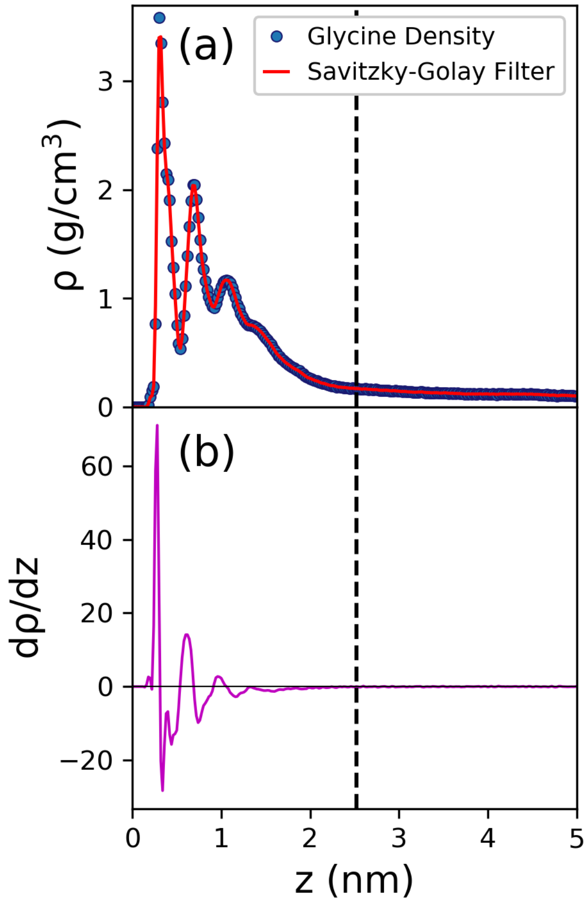


**Figure S11:** (a) Example of the Savitzky-Golay filter used to smooth the glycine density profile of the 500.7 g/kg, 6.1 nm film prior to taking the derivative. (b) The derivative of the smoothed density profile. The dashed black line represents the point where the derivative crosses the $x$-axis after the initial peaked area, defining the interfacial region.

## S8 References


[1] Junmei Wang, Romain M Wolf, James W Caldwell, Peter A Kollman, and David A Case. Development and testing of a general amber force field. *Journal of computational chemistry*, 25(9):1157–1174, 2004.

[2] JL Derissen, PH Smit, and J Voogd. Calculation of the electrostatic lattice energies of. alpha.-,. beta.-, and. gamma.-glycine. *The Journal of Physical Chemistry*, 81(15):1474–1476, 1977.

[3] HJC Berendsen, JR Grigera, and TP Straatsma. The missing term in effective pair potentials. *Journal of Physical Chemistry*, 91(24):6269–6271, 1987.

[4] Alexei M Nikitin, Yury V Milchevskiy, and Alexander P Lyubartsev. A new amber-compatible force field parameter set for alkanes. *Journal of molecular modeling*, 20(3):2143, 2014.

[5] Junmei Wang, Wei Wang, Peter A Kollman, and David A Case. Antechamber: an accessory software package for molecular mechanical calculations. *J. Am. Chem. Soc*, 222:U403, 2001.

[6] Abraham Savitzky and Marcel JE Golay. Smoothing and differentiation of data by simplified least squares procedures. *Analytical chemistry*, 36(8):1627–1639, 1964.
